# Changes in Metabolites During an Oral Glucose Tolerance Test in Early and Mid-Pregnancy: Findings from the PEARLS Randomized, Controlled Lifestyle Trial

**DOI:** 10.3390/metabo10070284

**Published:** 2020-07-10

**Authors:** Danielle E. Haslam, Jun Li, Liming Liang, Marijulie Martinez, Cristina Palacios, Maria A. Trak-Fellermeier, Paul W. Franks, Kaumudi Joshipura, Shilpa N. Bhupathiraju

**Affiliations:** 1 Channing Division of Network Medicine, Department of Medicine, Brigham and Women’s Hospital, 75 Francis Street, Boston, MA 02115, USA; sbhupath@hsph.harvard.edu; 2 Harvard Medical School, 25 Shattuck St, Boston, MA 02115, USA; 3 Department of Nutrition, Harvard T.H. Chan School of Public Health, 655 Huntington Ave, Boston, MA 02115, USA; junli@hsph.harvard.edu; 4 Department of Epidemiology, Harvard T.H. Chan School of Public Health, 655 Huntington Ave, Boston, MA 02115, USA; lliang@hsph.harvard.edu (L.L.); kaumudi.joshipura@upr.edu (K.J.); 5 Department of Biostatistics, Harvard T.H. Chan School of Public Health, 655 Huntington Ave, Boston, MA 02115, USA; 6 Center for Clinical Research and Health Promotion, University of Puerto Rico Medical Sciences Campus, San Juan, PR 00936-5067, Puerto Rico; marijulie.martinez@upr.edu; 7 Dietetics and Nutrition Department, Robert Stempel College of Public Health & Social Work, Florida International University, 11200 SW 8th Street AHC5, Miami, FL 33199, USA; cristina.palacios@upr.edu (C.P.); mtrakfel@fiu.edu (M.A.T.-F.); 8 Lund University Diabetes Centre, CRC, SUS Malmö, Jan Waldenströms gata 35, House 91:12, SE-214 28 Malmö, Sweden; paul.franks@med.lu.se

**Keywords:** metabolomics, OGTT, GDM, omics, pregnancy, diet, physical activity

## Abstract

The oral glucose tolerance test (OGTT) is used to diagnose gestational and other types of diabetes. We examined metabolite changes during an OGTT, and how a comprehensive diet and physical activity intervention may influence these changes in a population of overweight/obese Hispanic pregnant women. Integration of changes in metabolites during an OGTT may help us gain preliminary insights into how glucose metabolism changes during pregnancy. Among women from the Pregnancy and EARly Lifestyle improvement Study (PEARLS), we measured metabolites during a multipoint OGTT (fasting, 30, 60 and 120 min) at early and mid-pregnancy. Metabolite levels were measured by liquid chromatography–mass spectrometry in plasma samples in the lifestyle intervention (*n* = 13) and control (*n* = 16) arms of the study. A total of 65 candidate metabolites were selected that displayed changes during an OGTT in previous studies. Paired and unpaired *t*-tests were used to examine differences in Δfast-120 min: (1) at early and mid-pregnancy; and (2) by intervention assignment. We applied principal component analysis (PCA) to identify those metabolites that differed by intervention assignment and OGTT time points. Most of the characteristic changes in metabolites post-OGTT were similar at both gestational time points. PCA identified characteristic metabolite patterns associated with OGTT time points at both early and mid-pregnancy. These metabolites included ketone bodies, tryptophan, acyl carnitines, polyunsaturated fatty acids, and biomarkers related to bile acid, urea cycle, arginine, and proline metabolism. PCA identified distinct Δfast-120 min in fatty acid, acyl carnitine, bile acid, ketone body, and amino acid levels at mid- compared to early pregnancy. Participants in the intervention group did not display mean decreases in Δfast-120 min of several long-chain acyl carnitines that were observed in the control group. These findings provide preliminary insight into metabolites, whose role in increased insulin resistance during pregnancy, should be explored further in future studies.

## 1. Introduction

Impaired glucose metabolism is a common condition that has a strong association with chronic diseases such as type 2 diabetes (T2D), cardiovascular disease, and cancer in the general population [1,2,3]. In pregnant women, one complication of impaired glucose metabolism is gestational diabetes mellitus (GDM) which, if untreated, can cause serious harm to the mother and fetus. GDM has been associated with a variety of adverse pregnancy and perinatal outcomes, and long-term health effects, including increased risk of developing T2D for women and increased rates of adiposity and disorders related to glucose metabolism in the offspring [4,5]. The prevalence of GDM is increasing, with 6–12% of pregnancies affected globally [6].

During a normal pregnancy, several changes in glucose and insulin metabolism lead to increased insulin resistance. These normal changes are driven by hormonal changes that spare carbohydrates as fuel for the fetus [7]. However, when combined with underlying chronic insulin resistance present in some women, this increased insulin resistance during pregnancy can lead to the development of GDM [8]. Although the mechanisms leading to increased insulin resistance during pregnancy are still not well-understood, female sex hormones and pregnancy-specific hormones secreted by the placenta, such as placental lactogen and growth hormone, likely drive systemic changes that influence insulin secretion and resistance [9]. The oral glucose tolerance test (OGTT) is an important tool for the diagnosis of both GDM and T2D that reflects glucose and insulin metabolism [10]. Integration of metabolic evaluation before and during an OGTT may help elucidate some of the mechanisms underlying changes in glucose and insulin metabolism during pregnancy.

In this study, we examined differences in plasma metabolites during an OGTT among pregnant women of Hispanic descent during early and mid-pregnancy. We also evaluated how changes in metabolites during an OGTT were influenced by a diet and lifestyle intervention, which was designed to achieve gestational weight gain within the Institute of Medicine guidelines [11] and improve metabolic outcomes.

## 2. Results

Table 1 describes the characteristics of participants in the Pregnancy and EARly Lifestyle improvement Study (PEARLS; ClinicalTrials.gov: NCT01771133) randomized trial by intervention group. The mean age at randomization was lower in the control arm (25.6 (±4.8) years) compared to the intervention arm (30.7 (±5.3) years). Body mass index (BMI) (±SD) was similar by intervention arm at 34.2 (±7.5) kg/m^2^ among women in the intervention arm and 36.2 (±6.8) kg/m^2^ in the control arm, respectively. Additional sociodemographic and lifestyle measures were also similar by intervention arm.

### 2.1. Influence of Intervention on Changes in Metabolites during an OGTT Among Pregnant Women

**Figure 1:**
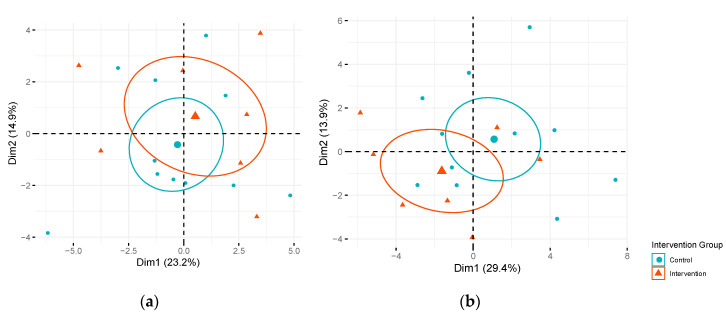
Score plot of principal component (PC) 1 versus PC2 by intervention arm. Fasting metabolites in intervention and control arms among Pregnancy and EARly Lifestyle improvement Study (PEARLS) participants: (**a**) during early (*n* = 29) and (**b**) mid-pregnancy (*n* = 18). Dimension 1 (Dim1) represents PC1 and Dimension 2 (Dim2) represents PC2. Percentage of variation in fasting metabolite levels explained by each PC is presented in parentheses. Ellipses represent 95% confidence ellipses by intervention group and larger symbols represent the center of the ellipse.

In analyses focused on individual candidate metabolites, two long-chain monounsaturated acyl carnitines (myristoleoylcarnitine (C14:1)* and palmitoleoylcarnitine (C16:1)*) displayed significant differences (False Discovery Rate (FDR) adjusted *p* < 0.05) in changes in metabolite levels from fasting to 120 min post oral glucose challenge (Δfast-120 min) in the intervention compared to the control arm at mid-pregnancy after FDR correction (Table 2). The Δfast-120 min for myristoleoylcarnitine (C14:1)* and palmitoleoylcarnitine (C16:1)* observed in the intervention arm at mid-pregnancy tended to be more similar to the Δfast-120 min observed during early pregnancy. No other significant differences in Δfast-120 min of other metabolites were observed in the intervention compared to the control arm during mid-pregnancy, but additional metabolites displayed suggestive differences in Δfast-120 min (unadjusted *p* < 0.05). These suggestive metabolites included acyl carnitines, amino acids, 3-hydroxybutyrate (BHBA), and glycodeoxycholate. As expected, no significant differences in Δfast-120 min were observed in the intervention compared to the control group for the OGTT conducted prior to randomization during early pregnancy. These results suggest that the intervention may have had some influence on changes in metabolites during the OGTT, but our sample size was too small to detect a statistical difference in these Δfast-120 min the intervention versus control arm for many metabolites. Figure 1 presents a principle component analysis (PCA) focused on fasting candidate metabolite levels colored by intervention or control arm during early pregnancy (Figure 1a) and mid-pregnancy (Figure 1b) by intervention assignment. The linear combinations of the most discriminating fasting metabolites were not significantly associated with intervention versus control during early or mid-pregnancy (Tables S1 and S2).

### 2.2. Changes in Metabolites during an OGTT

**Figure 2:**
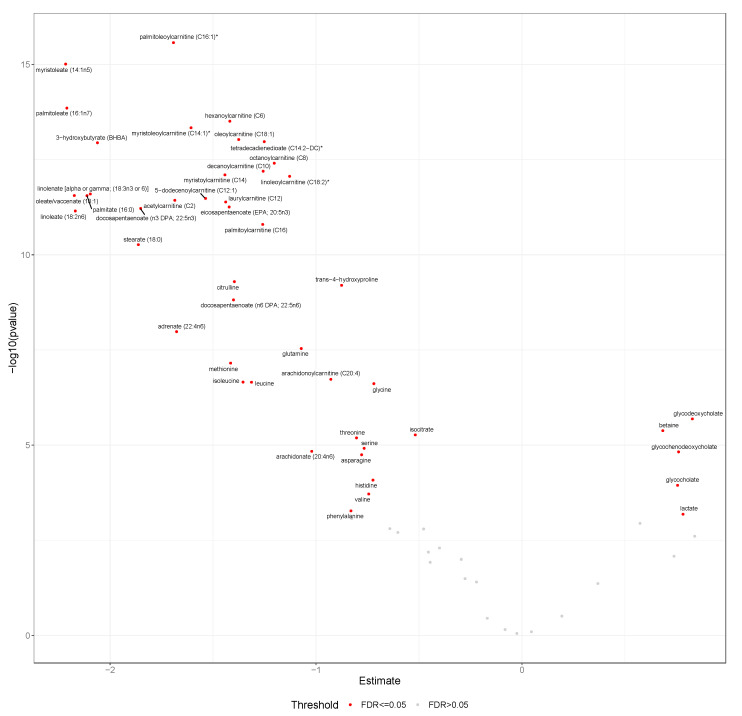
Mean Δfast-120 min for metabolites at early pregnancy. This volcano plot displays mean Δfast-120 min post-OGTT and -log_10_ *p* values for all candidate metabolites (*n* = 65) among PEARLS participants at the baseline visit (*n* = 29). Metabolites labeled with red passed the false discovery rate threshold of *p* < 0.05. Abbreviations: FDR, false discovery rate; OGTT, oral glucose tolerance test; PEARLS, Pregnancy and EARly Lifestyle improvement Study; Δfast-120 min, changing in glucose from fasting to 120 min during the OGTT.

We found that 47 of the 65 selected candidate metabolites displayed significant differences (FDR-adjusted *p* < 0.05) between Δfast-120 min during early pregnancy (baseline) (Figure 2), and 45 during mid-pregnancy (about 36 weeks) (Figure S1). Twelve of the candidate metabolites (18%) did not display significant differences in Δfast-120 min during either early or mid-pregnancy, while an additional six did not display significant differences during early pregnancy (9%) and eight during mid-pregnancy (12%).

The majority of the metabolites that decreased in response to the glucose load included those involved in fatty acid and amino acid metabolism (Table S3). Fatty acids included long-chain monounsaturated, polyunsaturated, and saturated fatty acids, acyl carnitines of varying chain length and saturation, and tetradecadienedioate (a dicarboxylate). Amino acid metabolites that decreased were related to metabolism of arginine, proline, lysine, leucine, isoleucine, valine, glycine, glutamine, serine, threonine, methionine, phenylalanine, histidine, alanine, and aspartate. Additional metabolites displaying relative decreases after the oral glucose load included 3-hydroxybutyrate, a ketone body, and isocitrate, a tricarboxylic acid cycle metabolite. Fewer metabolites increased post-OGTT, but we observed relative increases in gluconate, lactate, and bile acids (glycocholate, glycochenodeoxycholate, glycodeoxycholate).

### 2.3. Differences in Changes in Metabolites during OGTT at Early Versus Mid-Pregnancy

**Figure 3:**
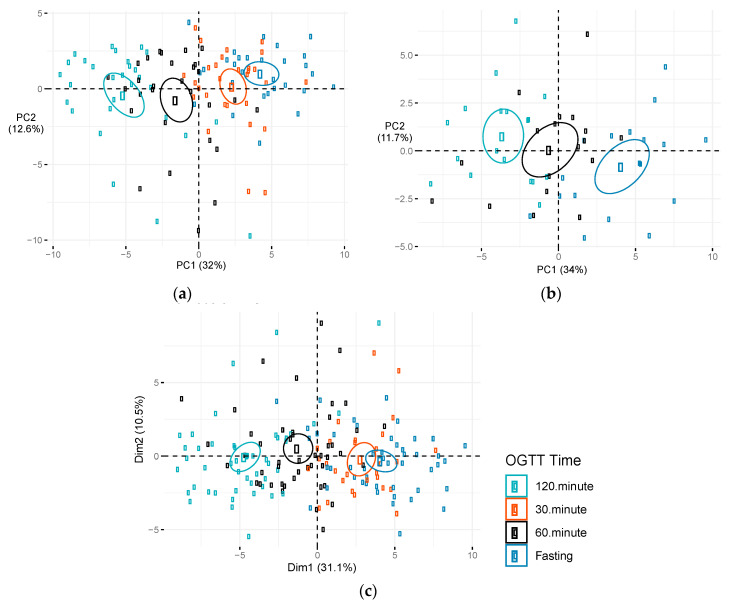
Score plot of principal component (PC) 1 versus PC2 by OGTT time. Metabolite levels during an OGTT among PEARLS participants: (**a**) During early pregnancy (*n* = 29), (**b**) mid-pregnancy (*n* = 18), and (**c**) combination of OGTTs conducted during early and mid-pregnancy. Figures depict plots of first two components. Dim1 represents PC1 and dim2 represents PC2. Percentage of variation in metabolite levels during an OGTT explained by each PC is presented in parentheses. Ellipses represent 95% confidence ellipses by OGTT time. Abbreviations: Dim, dimension; OGTT, oral glucose tolerance test; PC, principal component.

The metabolites displaying significant changes after OGTT were similar at early- and mid-pregnancy (Table S3). Several metabolites displayed suggestive differences in Δfast-120 min between early and mid-pregnancy, but none were statistically significant after FDR correction for multiple testing in single metabolite analyses. Metabolites displaying suggestive differences included long chain fatty acids (monounsaturated, n-3 and n-6 polyunsaturated, and saturated), tetradecadienedioate, acyl carnitines, ketone bodies, and metabolites related to the urea cycle and amino acid metabolism. PCA of changes in metabolites (Δfast- 120min) for all OGTT (conducted at both early and mid-pregnancy) suggested that principle component (PC) 2, but not PC1, significantly predicted whether the OGTT was conducted at early or mid-pregnancy (Figure S2). PC2 explained 18.3% of the variability in Δfast-120 min metabolite changes and was negatively associated with time the OGTT was conducted during pregnancy (*p* = 0.01; Table S4). The largest weights for PC2 included positive weights for fructose, bile acids, and amino acids and negative weights for long chain fatty acids (monounsaturated, n-3 and n-6 polyunsaturated, and saturated), acyl carnitines, tetradecadienedioate, and ketone bodies (Table S5). Many of the metabolites that had the largest weights in the linear combination defining PC2 also displayed suggestive differences in the single metabolite analyses. Given that changes in metabolites during the OGTT did not differ substantially in the intervention and control arms, we combined these groups and used PCA to identify patterns of metabolites associated with OGTT time points during early and mid-pregnancy. Figure 3 plots the first two principal components identified in the PCA of candidate metabolites during early pregnancy (Figure 3a), mid-pregnancy (Figure 3b), and the combination of early and mid-pregnancy (Figure 3c). Each data point is colored by the time that the metabolite was measured during the OGTT (fasting and 30, 60, and 120 min post-OGTT). High discrimination by OGTT time points was observed at both early and mid-pregnancy. The first principal component (PC1), was significantly associated with OGTT time points during early pregnancy, mid-pregnancy and when OGTTs were combined at early and mid-pregnancy (Table 3). Table S5 presents factor loadings for PC1, which accounted for >30% of the variation in metabolite levels at the OGTT conducted during early and mid-pregnancy. PC1 was heavily weighted on relative decreases in acyl carnitines, fatty acids (monounsaturated, saturated, and polyunsaturated), ketone bodies, and metabolites related to leucine, isoleucine, and valine metabolism. The metabolite weights in the second principal component (PC2) for early and mid-pregnancy differed substantially in their factor loadings (data not shown), and PC2 was not significantly associated with OGTT time points at early or mid-pregnancy (Table 3).

### 2.4. Sensitivity Analyses

Changes in glucose concentrations during OGTT by time during pregnancy (Figure S3) and intervention status (Figure S4) were similar, suggesting that the further investigation into changes in metabolites in the current study is warranted. Associations between PC’s and OGTT time points and intervention versus control arm were similar when all metabolites were included (*n* = 883) (data not shown). Sensitivity analyses were also performed adjusting for age (Figure S5) and excluding participants who reported drinking alcohol during pregnancy, those who developed GDM, and those with baseline hypertension yielded similar results for all analyses (data not shown).

## 3. Discussion

In this analysis among pregnant women, we observed relative decreases in acyl carnitines, ketone bodies, fatty acids, amino acids, and isocitrate and increases in bile acids, lactate and gluconate from fasting to 120 min following a 75 g glucose load at early/mid-pregnancy. Most changes in metabolites were similar during early- and mid-pregnancy, but PCA revealed characteristic patterns of changes in long chain fatty acids (monounsaturated, n-3 and n-6 polyunsaturated, saturated), acyl carnitines, ketone bodies, bile acids and amino acids during the OGTT that were significantly different at early compared to mid-pregnancy. The lifestyle intervention designed to achieve appropriate gestational weight gain may help preserve characteristic metabolite changes during a standard OGTT that may be disrupted during mid-pregnancy, with the strongest evidence for long-chain acyl carnitine metabolites.

### 3.1. Changes in Metabolites during an OGTT at Early Versus Mid-Pregnancy

This is the first study, to date, examining whether changes in metabolites post-OGTT may be different during early versus mid-pregnancy. Several studies have examined metabolomic responses to an OGTT in the general population [12,13,14,15,16,17,18], and three studies have examined the response during mid-pregnancy in Peruvian [19] and European women [20]. Our findings, at around 36 weeks gestation, replicated relative decreases in acyl carnitines, amino acids, and fatty acids, observed in these studies, but we did not replicate the relative increases in bile acid metabolites observed in one study [19]. About 82% of the selected candidate metabolites that displayed significant changes during an OGTT in at least two previous studies [12,13,14,15,16,17,18,19,20,21,22] were associated with significant changes during OGTT at either early or mid-pregnancy in this study. This suggests remarkable consistency in the pattern of changes in metabolites post-OGTT among various populations. This also confirms findings from previous studies that suggest post-prandial metabolites may differ from those at fasting [12,13,14,15,16,19,20]. The candidate metabolites that did not display significant changes during the OGTT in individual association analyses and were not heavily weighted in the PCA in this population may likely be metabolites that differ by sex, pregnancy status, race, or disease status.

PCA identified a pattern of metabolites that differed during early versus mid-pregnancy. Changes in fatty acids, acyl carnitines, bile acids, BHBA, and amino acid levels 120 min post-OGTT were smaller during mid-pregnancy, compared to early pregnancy. Although these metabolite differences were not statistically significant in single metabolite analyses, this metabolic profile was significantly different during early versus mid-pregnancy. In previous studies, some of these metabolites have been linked to insulin resistance, pregnancy, or both [23,24,25,26,27,28,29,30,31]. Previous studies have observed relative decreases in fatty acids post-OGTT among pregnant women [19,20], but this is the first study to measure metabolites during both early and mid-pregnancy. Therefore, these metabolites that displayed suggestive differences in response to an OGTT warrant further investigation in larger studies. Studies suggest that acyl carnitines are tightly regulated, decrease in women during pregnancy [26,27], and have been associated with maternal and child overweight and obesity [28]. Docosapentaenoate (DPA), a fatty acid, has been inversely correlated with insulin resistance markers, and circulating levels tend to decrease during pregnancy [29]. Bile acids are tightly regulated during pregnancy and increases are indicative of intrahepatic cholestasis of pregnancy [30,31]. Bile acids have also been associated with non-alcoholic fatty liver disease [32], which is an independent risk factor for T2D [33]. BHBA has also been linked to both insulin resistance and macrosomia in previous studies [23,24,25]. Studies have consistently observed that dimethylarginine (ADMA) levels during pregnancy are elevated in those with GDM and ADMA, and has also been associated insulin resistance and several other cardiometabolic risk factors [34]. Our data indicate that changes in ADMA during an OGTT may be exacerbated during pregnancy, which could suggest ADMA plays a role in gestational insulin resistance. Although our results are not conclusive due to limited sample size, further investigation into the role these metabolites play in glucose metabolism during pregnancy is warranted.

### 3.2. Influence of Intervention on Changes in Metabolites during OGTT Among Pregnant Women

Our findings suggest that changes in acyl carnitines during an OGTT, particularly two long chain monounsaturated acyl carnitines (myristoleoylcarnitine (C14:1)* and palmitoleoylcarnitine (C16:1)*), were significantly different among participants who were part of the lifestyle intervention implemented in the PEARLS randomized lifestyle trial compared to participants in the control group. Both the single metabolite and PCA analyses also showed trends toward smaller decreases in changes in acyl carnitines, amino acids, BHBA, and glycodeoxycholate during the OGTT among the intervention arm compared to the control arm, but only changes in myristoleoylcarnitine (C14:1)* and palmitoleoylcarnitine (C16:1)* were statistically significant. Given the null findings in the primary intervention, which was not successful in achieving the gestational weight gain targets set by the Institute of Medicine in the PEARLS trial [35], these differences between the two arms are interesting to inform future studies. The results in the overall trial were suggestive of a benefit and other studies suggest that improved diet and physical activity are modifiable risk factors associated with proper gestational weight gain [36,37,38] and better glucose tolerance [39,40]. The intervention implemented here may not have been intensive enough to influence gestational weight gain targets, and compliance was also low, indicating that further intervention strategies are necessary. Future studies should examine metabolite changes during an OGTT in intervention studies that produce larger changes in gestational weight gain and maternal health during pregnancy, in order to aid in understanding the biological underpinnings of the intervention benefits. Our results suggest that changes in acyl carnitines, amino acids, BHBA, and glycodeoxycholate may be of particular interest for future studies.

### 3.3. Study Strengths and Limitations

Our study has several strengths and limitations that should be considered when interpreting our results. A major strength is that we have repeated measures of a large number of metabolites among the same individuals at several time points during two OGTTs. This enabled us to conduct paired statistical analyses that minimize confounding by participant characteristics. The lifestyle intervention was also implemented as part of a rigorously designed randomized, controlled trial with a low dropout rate. Given the small sample size, we focused on candidate metabolites, associated with OGTT time points in previous studies, hence, reducing the burden of multiple testing. Yet, we still adjusted for multiple testing using an FDR approach even for the candidate metabolites. A major limitation of our study is the small sample size with only few individuals developing GDM during the study. Due to limitations in the methods available for the measurement of metabolites, we were only able to examine relative levels of metabolites, as opposed to absolute concentrations. Given the limited sample size, our results should be considered hypothesis generating and should be interpreted with caution. These findings should be validated in future studies that also expand to pregnant women of different race/ethnicity and implement new interventions.

## 4. Materials and Methods

### 4.1. Study Population

This analysis used data collected as part of the PEARLS (ClinicalTrials.gov: NCT01771133), which is described in detail elsewhere [35]. Briefly, PEARLS is a behavioral and lifestyle randomized intervention trial, conducted among obese/overweight pregnant adult women from a median of 14.6 weeks (baseline) to 36 weeks gestational age, aged ≥18 years, living in Puerto Rico. The intervention was designed to increase adherence to the Institute of Medicine’s gestational weight gain guidelines [11], and to improve maternal and neonatal metabolic health. This study was conducted between January 2013 and August 2015 at the University Hospital in Puerto Rico. The diet and physical activity intervention was delivered within a health empowerment theoretical framework in both group and individual sessions that included tracking of diet, physical activity, and weight trajectory, with a primary focus on total calories [41]. Dietary guidelines included specific targets for food quantity and quality during each pregnancy phase, and encouragement to improve carbohydrate and fat quality, reduce salt, replace red meat with low-mercury fish, nuts and beans, and take multivitamins prescribed by their physicians. Physical activity guidelines encouraged daily exercise routines and a decrease in sedentary time. The control group received health advice unrelated to the study outcomes in group sessions. The primary outcome of the intervention trial was to achieve appropriate gestational weight gain and infant birth weight. PEARLS is part of the larger LIFE-Moms Consortium, which includes nine institutions that conducted a similar behavioral and lifestyle intervention with the same goals [42].

A total of 31 women completed the trial, and 2 women were excluded from the study due to missing metabolomics data. Among those with metabolomics data available, 13 women were in the intervention arm and 16 women were in the control arm. Sociodemographic characteristics, height, weight, and biological data were collected via examination and interviewer-administered questionnaires. Biological specimens were collected by a nurse at <16 weeks (baseline or early pregnancy) and 35–36 weeks of gestational age (mid-pregnancy). Women underwent a 2-hr, 75-g OGTT during early and mid-pregnancy, after a 12-h overnight fast. Blood samples were drawn at fasting, 30 min (early pregnancy only), 60 min, and 120 min, and were immediately processed. Plasma samples were kept at −80 °C until they were shipped on dry ice for metabolomic analysis. PEARLS was registered at ClinicalTrials.gov [NCT01771133] and was approved by the University of Puerto Rico Institutional Review Board. Women agreeing to partake in the study procedures for mother-infant dyads provided written informed consent.

### 4.2. Metabolomics Profiling

Plasma metabolomic profiling was performed using the Metabolon platform via ultra-high-performance liquid chromatography tandem mass spectrometry (UHPLC/MS/MS), resulting in the detection of 974 named metabolites. Quality control procedures were performed throughout, internal standards were included, and the injection order was random. We removed metabolites with a detection rate <75%, and any missing values were imputed with a value equal to half the minimum of each metabolite measure. Due to skewed distributions and differing scales, Z-scores were calculated to standardize and normalize each metabolite. After exclusions, a total of 883 named metabolites were available. Given that several previous studies have examined changes in metabolites during an OGTT among the general population and in pregnant women, we focused on 65 candidate metabolites that appeared to change during an OGTT in multiple previous studies (Table S5) [12,13,14,15,16,17,18,19,20,21,22].

### 4.3. Participant Characteristics

Gestational age at randomization (weeks and days) was clinically determined by last ultrasound [42]. Questionnaires were used to obtain maternal age, education level (six categories grouped into college education or high school education/diploma/less), total family income (14 categories grouped into ≤$9999, $10,000–$19,999, and ≥$20,000 per year), marital status (married/living with a partner or single/separated/divorced/widowed), current smoking status (yes/no), alcohol drinking (yes/no), and parity (parous/nonparous). Trained and certified staff measured maternal height and body weight. BMI was calculated by dividing a person’s weight (kg) by the square of their height (m). Women were clinically screened for GDM between 24 and 27 weeks of gestational age with a 2-h 75 g OGTT [43]. To estimate dietary consumption habits, trained interviewers administered a 193 item semi-quantitative food frequency questionnaire with food replicas and other visual aids that was adapted and validated for Puerto Ricans [44].

### 4.4. Statistical Analysis

Means ± standard deviations and N (%) for key baseline characteristics of participants were calculated separately in the intervention and the control group. First, we used Student’s paired *t*-tests to compare the mean difference in metabolites at fasting and 120 min for each OGTT individually (early and mid-pregnancy). Then, we calculated the change in standardized metabolite levels between Δfast-120 min during both early and mid-pregnancy. Student’s paired and unpaired *t*-tests were utilized to examine whether there were significant differences between Δfast-120 min: (1) during early and mid-pregnancy, and (2) by intervention arm, respectively. Plots were created to visualize mean Δfast-120 min and −log_10_ *p* values. *p* values were corrected for multiple testing using a FDR to achieve a global α = 0.05.

Metabolite profiles were derived using principal components analysis (PCA) for candidate metabolites (*n* = 65). For the top two principal components, accounting for the greatest amount of variability in the data, a weighted metabolite score was calculated for each individual during each OGTT or at each OGTT time point. We examined whether the PCA-derived linear combination of metabolite Δfast-120 min, during all OGTT, were associated with: (1) early or mid-pregnancy; or (2) intervention arm. Given that changes in metabolites during the OGTT were largely similar by intervention and control arms, we combined the metabolite levels at all OGTT time points, and used PCA to derive linear combinations of metabolites and evaluate their association with OGTT time points (fasting, 30 min, 60 min, and 120 min), regardless of intervention status and separated by time during pregnancy. A large number of metabolites were measured in addition to the selected candidate metabolites, so we also performed sensitivity analyses for all PCA, including all available named metabolites (*n* = 883). As a supplement to our analyses of changes in metabolites during the OGTT, we used Student’s *t*-tests to compare differences in Δfast-120 min of glucose concentrations for: (1) early versus mid-pregnancy; and (2) intervention versus control arm. Sensitivity analyses were also conducted adjusting for age and excluding participants who reported drinking alcohol during pregnancy (N = 1), those who developed GDM during pregnancy (N = 5), and those with baseline hypertension (N = 5). All statistical analyses were conducted using R (version 2.6.0) statistical software.

## 5. Conclusions and Future Perspectives

The suggestive changes in metabolites related to long chain fatty acids (monounsaturated, n-3 and n-6 polyunsaturated, saturated), acyl carnitines, ketone bodies, bile acids and amino acids during the OGTT at early versus mid-pregnancy in this study may be important candidate metabolites to target in future studies. Larger studies could examine the relationship between GDM development and concentrations of these metabolites’ pre-pregnancy and throughout pregnancy. Acyl carnitines may be of particular interest to further understand how their concentrations influence GDM risk among pregnant women, and how diet and lifestyle interventions may modify this risk. Integration of female sex hormone and placental hormone measurements with metabolite levels may also be useful in understanding how these metabolites may mediate changes in insulin resistance driven by pregnancy-related hormonal changes [45]. Future studies may also benefit from examining correlations between metabolite changes during an OGTT among pregnant women to identify how pathways may be perturbed.

We had too few GDM cases to examine the differences in metabolites by GDM status, but identification of new biomarkers of glucose metabolism among pregnant women may help generate hypotheses that could lead to a better understanding of the underlying mechanisms that lead to the development of GDM. Several indicators of GDM, such as inflammatory, lipid, epigenetic, and insulin sensitivity biomarkers have been explored in previous studies, but inconsistency in validity and reproducibility across populations has limited their clinical value [46]. Identification of metabolite concentrations or changes in concentrations that are indicative of higher risk of GDM may be important biomarkers contributing to earlier diagnoses of GDM and identify women who would benefit from early lifestyle intervention to reduce subsequent risk of GDM.

In summary, integration of metabolite levels with the typical measures of glucose concentrations during an OGTT identified several key metabolites where post-OGTT responses displayed suggestive differences during early- and mid-pregnancy, and due to the lifestyle intervention implemented in the PEARLS trial. These metabolites may be candidate metabolites for further investigation into mechanisms leading to increases in insulin resistance among pregnant women.

## Figures and Tables

**Table 1:** Characteristics of PEARLS participants during early pregnancy in the current study stratified by intervention arm, N = 29 ^1^.

Characteristic	Intervention (N = 13)	Standard Care (N = 16)
Age at randomization, years	* 30.7 ± 5.3	* 25.6 ± 4.8
Body mass index, kg/m^2^	34.2 ± 7.5	36.2 ± 6.8
Educational level		
High school education/diploma or less	6 (46)	8 (50)
College education	7 (54)	8 (50)
Total annual family income		
≤$9999	5 (38.5)	8 (50)
$10,000–$19,999	5 (38.5)	5 (31)
≥$20,000	3 (23)	3 (19)
Marital Status		
Married/living with a partner	8 (62)	14 (87.5)
Single/separated/divorced/widowed	5 (38)	2 (12.5)
Current Smoking	0 (0)	0 (0)
Any alcohol intake during pregnancy	1 (7.7)	0 (0)
Parous	9 (69)	11 (69)
Gestational Diabetes at 24 weeks	2 (15)	3 (19)
Fasting glucose (mg/dL)	* 95.5 ± 7.6	* 90.5 ± 4.5
Total energy intake, kcal/day ^2^	2501 ± 873	2523 ± 945

^1^ Values are n (percentage) or mean ± standard deviation. Student’s *t* tests were conducted, and * represents *p* < 0.05. ^2^ Only participants with a plausible total energy intake (>1075 kcal and <4800 kcal) were included (N = 26).

**Table 2:** Mean difference at fasting compared to 120 min post-OGTT (Δfast-120 min) for targeted metabolites in intervention versus control arm among PEARLS participants at early and mid-pregnancy.

Metabolite	HMDB ID	Biological Description	Early Pregnancy ^1^			Mid-Pregnancy ^2^		
			Intervention Mean (SD)	Control Mean (SD)	*p*-Value	Intervention Mean (SD)	Control Mean (SD)	*p*-Value
myristoleoylcarnitine (C14:1) *	NA	Fatty Acid Metabolism (Acyl Carnitine, Monounsaturated)	−1.7 (0.7)	−1.5 (0.5)	0.50	−1.7 (0.4)	−0.9 (0.4)	** 0.0003
palmitoleoylcarnitine (C16:1) *	HMDB00641	Fatty Acid Metabolism (Acyl Carnitine, Monounsaturated)	−1.6 (0.4)	−1.5 (0.5)	0.67	−1.8 (0.4)	−1.0 (0.4)	** 0.0008
**leucine**	NA	Leucine, Isoleucine and Valine Metabolism	−1.2 (1.0)	−1.6 (0.3)	0.31	−1.8 (0.2)	−1.3 (0.3)	0.004
linoleoylcarnitine (C18:2) *	HMDB06469	Fatty Acid Metabolism (Acyl Carnitine, Polyunsaturated)	−1.0 (0.5)	−1.2 (0.5)	0.51	−1.4 (0.6)	−0.6 (0.4)	0.009
**5-dodecenoylcarnitine (C12:1)**	HMDB13326	Fatty Acid Metabolism (Acyl Carnitine, Monounsaturated)	−1.7 (0.5)	−1.5 (0.8)	0.52	−1.7 (0.7)	−0.8 (0.4)	0.01
**myristoylcarnitine (C14)**	HMDB05066	Fatty Acid Metabolism (Acyl Carnitine, Long Chain Saturated)	−1.4 (0.6)	−1.3 (0.6)	0.68	−1.6 (0.5)	−0.9 (0.7)	0.02
**palmitoylcarnitine (C16)**	HMDB00222	Fatty Acid Metabolism (Acyl Carnitine, Long Chain Saturated)	−1.1 (0.5)	−1.2 (0.7)	0.60	−1.4 (0.7)	−0.6 (0.5)	0.02
**3-hydroxybutyrate (BHBA)**	HMDB00929	Ketone Bodies	−2.2 (0.6)	−2.0 (0.6)	0.40	−2.0 (0.6)	−1.3 (0.5)	0.02
**oleoylcarnitine (C18:1)**	HMDB05065	Fatty Acid Metabolism (Acyl Carnitine, Monounsaturated)	−1.2 (0.5)	−1.3 (0.7)	0.81	−1.5 (0.8)	−0.6 (0.4)	0.02
**asparagine**	HMDB00168	Alanine and Aspartate Metabolism	−0.7 (0.8)	−1.1 (0.6)	0.31	−1.4 (0.5)	−0.7 (0.7)	0.02
**glycodeoxycholate**	HMDB00631	Secondary Bile Acid Metabolism	0.5 (1.0)	0.8 (0.6)	0.53	0.6 (0.4)	−0.04 (0.7)	0.03
**tryptophan**	HMDB00357	Tryptophan Metabolism	−0.3 (0.9)	−0.9 (0.7)	0.23	−0.5 (0.4)	−0.9 (0.4)	0.03
**glutamine**	NA	Glutamate Metabolism	−1.2 (0.4)	−1.1 (0.8)	0.62	−1.2 (0.4)	−0.7 (0.6)	0.04
**adrenate (22:4n6)**	HMDB02226	Long Chain Polyunsaturated Fatty Acid (n3 and n6)	−2.2 (1.3)	−1.6 (0.8)	0.24	−1.7 (0.3)	−0.9 (1.2)	0.07
**isoleucine**	HMDB00172	Leucine, Isoleucine and Valine Metabolism	−1.4 (0.9)	−1.6 (0.3)	0.56	−1.7 (0.3)	−1.4 (0.3)	0.07
**linoleate (18:2n6)**	HMDB00673	Long Chain Polyunsaturated Fatty Acid (n3 and n6)	−2.4 (1.3)	−2.2 (0.5)	0.61	−2.0 (0.5)	−1.4 (0.8)	0.08
**ornithine**	HMDB03374	Urea cycle; Arginine and Proline Metabolism	−0.4 (0.8)	−0.3 (0.5)	0.85	−0.7 (0.4)	−0.4 (0.5)	0.08
**glycocholate**	HMDB00138	Primary Bile Acid Metabolism	0.8 (0.6)	0.6 (0.7)	0.51	0.5 (0.8)	−0.2 (0.6)	0.09
**linolenate [alpha or gamma; (18:3n3 or 6)]**	HMDB03073	Long Chain Polyunsaturated Fatty Acid (n3 and n6)	−2.4 (1.1)	−2.1 (0.5)	0.63	−1.9 (0.4)	−1.4 (0.8)	0.09
**pyruvate**	HMDB00243	Glycolysis, Gluconeogenesis, and Pyruvate Metabolism	0.7 (1.2)	0.5 (0.9)	0.81	0.6 (0.6)	0.1 (0.5)	0.09
**hexanoylcarnitine (C6)**	HMDB00705	Fatty Acid Metabolism (Acyl Carnitine, Medium Chain)	−1.5 (0.4)	−1.6 (0.5)	0.66	−1.4 (0.5)	−1.0 (0.5)	0.10
**oleate/vaccenate (18:1)**	NA	Long Chain Monounsaturated Fatty Acid	−2.4 (1.1)	−2.2 (0.6)	0.66	−2.0 (0.6)	−1.4 (0.8)	0.10
**acetylcarnitine (C2)**	HMDB00201	Fatty Acid Metabolism (Acyl Carnitine, Short Chain)	−1.6 (0.6)	−1.6 (0.6)	0.93	−1.5 (0.5)	−1.2 (0.4)	0.12
**glycochenodeoxycholate**	HMDB00637	Primary Bile Acid Metabolism	1.0 (1.0)	0.8 (0.7)	0.62	0.6 (0.5)	0.1 (0.7)	0.12
**palmitoleate (16:1n7)**	HMDB03229	Long Chain Monounsaturated Fatty Acid	−2.4 (1.2)	−2.2 (0.6)	0.65	−1.8 (0.5)	−1.4 (0.6)	0.12
**laurylcarnitine (C12)**	HMDB02250	Fatty Acid Metabolism (Acyl Carnitine, Medium Chain)	−1.6 (0.6)	−1.5 (0.8)	0.87	−1.2 (0.6)	−0.8 (0.4)	0.13
tetradecadienedioate (C14:2-DC) *	NA	Fatty Acid, Dicarboxylate	−1.7 (0.5)	−1.1 (0.4)	0.02	−1.0 (0.2)	−0.7 (0.5)	0.13
**myristoleate (14:1n5)**	HMDB02000	Long Chain Monounsaturated Fatty Acid	−2.3 (0.9)	−2.2 (0.7)	0.77	−1.8 (0.6)	−1.3 (0.7)	0.17
**valine**	HMDB00883	Leucine, Isoleucine and Valine Metabolism	−0.5 (0.8)	−1.1 (0.6)	0.15	−1.1 (0.4)	−0.8 (0.6)	0.18
**methionine**	HMDB00696	Methionine, Cysteine, SAM and Taurine Metabolism	−1.3 (1.0)	−1.7 (0.6)	0.27	−1.7 (0.4)	−1.4 (0.4)	0.19
**lactate**	HMDB00190	Glycolysis, Gluconeogenesis, and Pyruvate Metabolism	0.9 (1.2)	0.7 (1.4)	0.70	0.3 (0.8)	0.8 (1.0)	0.21
**decanoylcarnitine (C10)**	HMDB00651	Fatty Acid Metabolism (Acyl Carnitine, Medium Chain)	−1.3 (0.4)	−1.3 (0.5)	0.92	−1.1 (0.7)	−0.7 (0.6)	0.21
**dimethylarginine (SDMA + ADMA)**	HMDB01539	Urea cycle; Arginine and Proline Metabolism	−0.3 (0.8)	−0.6 (0.8)	0.57	−1.2 (0.3)	−0.9 (0.5)	0.22
**octanoylcarnitine (C8)**	HMDB00791	Fatty Acid Metabolism (Acyl Carnitine, Medium Chain)	−1.3 (0.4)	−1.3 (0.5)	0.98	−1.1 (0.6)	−0.7 (0.5)	0.23
**alanine**	HMDB00161	Alanine and Aspartate Metabolism	0.3 (0.8)	−0.3 (0.7)	0.11	0.1 (0.6)	−0.2 (0.5)	0.24
**arginine**	HMDB00517	Urea cycle; Arginine and Proline Metabolism	−0.2 (1.4)	−0.9 (0.6)	0.27	−0.4 (0.7)	−0.8 (0.6)	0.25
**phenylalanine**	HMDB00159	Phenylalanine Metabolism	−0.7 (1.4)	−1.2 (0.6)	0.45	−1.4 (0.4)	−1.1 (0.6)	0.25
**docosapentaenoate (n6 DPA; 22:5n6)**	HMDB01976	Long Chain Polyunsaturated Fatty Acid (n3 and n6)	−2.0 (0.7)	−1.3 (0.5)	0.06	−1.3 (0.5)	−1.0 (0.8)	0.33
**isocitrate**	HMDB00193	TCA Cycle	−0.6 (0.6)	−0.6 (0.5)	0.89	−0.2 (0.4)	0.2 (1.1)	0.35
**threonine**	HMDB00167	Glycine, Serine and Threonine Metabolism	−0.6 (0.8)	−1.1 (0.4)	0.21	−0.9 (0.3)	−0.8 (0.2)	0.37
**eicosapentaenoate (EPA; 20:5n3)**	HMDB01999	Long Chain Polyunsaturated Fatty Acid (n3 and n6)	−1.7 (0.7)	−1.3 (0.6)	0.32	−1.5 (0.5)	−1.2 (0.9)	0.37
**lysine**	HMDB00182	Lysine Metabolism	−0.5 (0.6)	−0.5 (0.6)	0.90	−0.8 (0.3)	−0.7 (0.4)	0.39
**uridine**	HMDB00296	Pyrimidine Metabolism, Uracil containing	−0.2 (0.7)	0.6 (0.6)	0.03	0.4 (0.5)	0.1 (0.7)	0.39
**palmitate (16:0)**	HMDB00220	Long Chain Saturated Fatty Acid	−2.4 (1.2)	−2.1 (0.6)	0.58	−1.8 (0.7)	−1.5 (0.9)	0.41
**glycine**	HMDB00123	Glycine, Serine and Threonine Metabolism	−0.4 (0.8)	−0.8 (0.5)	0.28	−0.9 (0.6)	−0.7 (0.8)	0.45
**stearoylcarnitine (C18)**	HMDB00848	Fatty Acid Metabolism (Acyl Carnitine, Long Chain Saturated)	−0.5 (0.8)	−0.6 (1.2)	0.78	−0.6 (0.8)	−0.3 (0.7)	0.46
**betaine**	HMDB00043	Glycine, Serine and Threonine Metabolism	1.1 (0.7)	0.5 (0.6)	0.08	0.1 (0.4)	0.3 (0.6)	0.46
**stearate (18:0)**	HMDB00827	Long Chain Saturated Fatty Acid	−2.1 (1.1)	−1.9 (0.5)	0.80	−1.7 (1.1)	−1.3 (1.0)	0.47
**2-aminoadipate**	HMDB00510	Lysine Metabolism	−0.7 (0.7)	−0.1 (1.1)	0.20	−0.6 (1.0)	−0.2 (0.9)	0.50
**aspartate**	HMDB00191	Alanine and Aspartate Metabolism	0.3 (1.4)	0.001 (0.8)	0.60	−0.3 (0.8)	−0.1 (0.8)	0.51
**quinolinate**	HMDB00232	Nicotinate and Nicotinamide Metabolism	−0.1 (0.6)	−0.3 (0.6)	0.54	−0.4 (0.3)	−0.3 (0.4)	0.52
**histidine**	HMDB00177	Histidine Metabolism	−0.8 (0.7)	−1.0 (0.8)	0.51	−1.1 (0.7)	−0.9 (0.3)	0.54
**docosapentaenoate (n3 DPA; 22:5n3)**	HMDB06528	Long Chain Polyunsaturated Fatty Acid (n3 and n6)	−2.4 (0.9)	−1.8 (0.6)	0.18	−1.6 (0.6)	−1.4 (0.8)	0.55
**citrulline**	HMDB00094	Urea cycle; Arginine and Proline Metabolism	−1.5 (0.4)	−1.4 (0.7)	0.78	−1.6 (0.7)	−1.4 (0.5)	0.59
**urate**	HMDB00289	Purine Metabolism, (Hypo)Xanthine/Inosine containing	−0.1 (0.4)	−0.3 (0.6)	0.35	−0.1 (0.7)	−0.2 (0.6)	0.64
**fructose**	HMDB00660	Fructose, Mannose and Galactose Metabolism	1.1 (1.3)	0.2 (1.8)	0.21	0.8 (0.8)	0.6 (1.0)	0.64
**tyrosine**	HMDB00158	Tyrosine Metabolism	−1.0 (1.4)	−1.0 (0.8)	0.98	−0.9 (1.1)	−1.1 (1.0)	0.66
**proline**	HMDB00162	Urea cycle; Arginine and Proline Metabolism	−0.2 (1.0)	−0.8 (0.5)	0.17	−0.7 (0.3)	−0.7 (0.2)	0.68
**hypoxanthine**	HMDB00157	Purine Metabolism, (Hypo)Xanthine/Inosine containing	1.4 (1.7)	0.5 (1.5)	0.30	0.2 (0.8)	0.02 (1,0)	0.69
**arachidonate (20:4n6)**	HMDB01043	Long Chain Polyunsaturated Fatty Acid (n3 and n6)	−1.4 (1.1)	−1.0 (0.7)	0.37	−1.1 (0.8)	−0.9 (1.0)	0.75
**taurine**	HMDB00251	Methionine, Cysteine, SAM and Taurine Metabolism	0.4 (1.3)	0.3 (0.8)	0.85	−0.1 (1.2)	0.1 (1.1)	0.78
**trans-4-hydroxyproline**	HMDB00725	Urea cycle; Arginine and Proline Metabolism	−0.8 (0.3)	−0.9 (0.4)	0.65	−0.4 (0.6)	−0.4 (0.5)	0.80
**glutamate**	HMDB00148	Glutamate Metabolism	0.1 (1.3)	−0.2 (0.9)	0.59	−0.3 (0.9)	−0.3 (0.8)	0.87
**serine**	HMDB00187	Glycine, Serine and Threonine Metabolism	−0.6 (1.1)	−0.8 (0.7)	0.64	−0.9 (1.0)	−1.0 (0.6)	0.89
**arachidonoylcarnitine (C20:4)**	NA	Fatty Acid Metabolism (Acyl Carnitine, Polyunsaturated)	−0.6 (0.4)	−0.9 (0.9)	0.39	−0.8 (1.0)	−0.7 (0.6)	0.91

^1^ Values represent mean changes in metabolites at fasting versus 120 min at the OGTT conducted at early pregnancy (N = 29; baseline OGTT prior to randomization), and *p*-values represent Student *t* tests comparing intervention to the control group without FDR correction. ^2^ Values represent mean changes in metabolites at fasting versus 120 min at the OGTT conducted at mid-pregnancy (about 36 weeks) (N = 18), and *p*-values represent Student *t* tests comparing intervention to the control group without FDR correction. ** Indicates statistically significant finding after FDR correction for multiple testing. Abbreviations: FDR, false discovery rate; OGTT, oral glucose tolerance test; SD, standard deviation.

**Table 3:** Mean difference in top principal component (PC) scores for targeted metabolites by OGTT time points at early (*n* = 29), mid-pregnancy (*n* = 18), and all oral glucose tolerance tests (OGTT) combined (*n* = 47) ^1^.

Fasting		30 min		60 min		120 min	
		β (SE)	*p* Value	β (SE)	*p* Value	β (SE)	*p* Value
Early pregnancy							
PC1	Ref.	−1.9 (0.7)	0.005	−5.8 (0.7)	<0.001	−9.4 (0.7)	<0.001
PC2	Ref.	−0.9 (0.7)	0.24	−1.8 (0.7)	0.02	−1.4 (0.7)	0.05
Mid-Pregnancy							
PC1	Ref.	-	-	−4.7 (1.0)	<0.001	−7.7 (1.0)	<0.001
PC2	Ref.	-	-	0.9 (0.8)	0.29	1.6 (0.8)	0.06
Early and Mid-Pregnancy							
PC1	Ref.	−1.2 (0.6)	0.04	−5.4 (0.5)	<0.001	−8.8 (0.5)	<0.001
PC2	Ref.	0.1 (0.6)	0.85	0.8 (0.5)	0.10	0.3 (0.5)	0.60

^1^ PC’s were derived from the 65 candidate metabolites measured during the OGTTs for all participants (metabolite levels at fasting and 30, 60, and 120 min post OGTT).

## Supplementary Materials

The following are available online at https://www.mdpi.com/2218-1989/10/7/284/s1, Figure S1: Mean Δfast-120 min for metabolites during mid-pregnancy, Figure S2: Score plot of principle component (PC) 1 versus PC2 for changes in metabolites from fasting to 120 min by pregnancy time, Figure S3. Changes in glucose during an oral glucose tolerance test (OGTT) at early and mid-pregnancy by participant in the Pregnancy and EARly Lifestyle improvement Study (PEARLS), Figure S4. Changes in glucose during an oral glucose tolerance test (OGTT) by intervention status at mid-pregnancy and by participants of the Pregnancy and EARly Lifestyle improvement Study (PEARLS), Figure S5: Mean Δfast-120 min for metabolites at early pregnancy adjusting for maternal age., Table S1: Mean difference in top principal component (PC) scores for fasting metabolites at baseline (*n* = 29) and mid-pregnancy (*n* = 18) in intervention group compared to the control group among PEARLS participants, Table S2, Factor loadings for linear combination of metabolites defining principal component 1 (PC1) for fasting metabolites at mid-pregnancy among Pregnancy and EARly Lifestyle improvement Study (PEARLS) participants, Table S3. Mean change in metabolites from fasting to 120 min post-OGTT (Δfast-120 min) for targeted metabolites during early versus mid-pregnancy among PEARLS participants, Table S4: Mean difference in top principal component (PC) scores for changes in metabolites from fasting to 120 min (Δfast-120 min) during early compared to mid-pregnancy (*n* = 18) (targeted metabolites), Table S5. Factor loadings from principal component (PC) analysis at early and mid-pregnancy.

## References

[1] American Cancer Society (2020). Cancer facts and figures. Https://www.cancer.org/content/dam/cancer-org/research/cancer-facts-and-statistics/annual-cancer-facts-and-figures/2020/cancer-facts-and-figures-2020.pdf.

[2] Barr E.L., Zimmet P.Z., Welborn T.A., Jolley D., Magliano D.J., Dunstan D.W., Cameron A.J., Dwyer T., Taylor H.R., Tonkin A.M. (2007). Risk of Cardiovascular and All-Cause Mortality in Individuals With Diabetes Mellitus, Impaired Fasting Glucose, and Impaired Glucose Tolerance. Circulation.

[3] (1999). Glucose tolerance and mortality: Comparison of WHO and American Diabetic Association diagnostic criteria. Lancet.

[4] Bellamy L., Casas J.-P., Hingorani A.D., Williams D. (2009). Type 2 diabetes mellitus after gestational diabetes: A systematic review and meta-analysis. Lancet.

[5] Szmuilowicz E.D., Josefson J.L., Metzger B.E. (2019). Gestational Diabetes Mellitus. Endocrinol. Metab. Clin. N. Am..

[6] Zhu Y., Zhang C. (2016). Prevalence of Gestational Diabetes and Risk of Progression to Type 2 Diabetes: A Global Perspective. Curr. Diabetes Rep..

[7] Homko C.J., Sivan E., Reece E.A., Boden G. (1999). Fuel metabolism during pregnancy. Semin. Reprod. Endocrinol..

[8] Barbour L.A., McCurdy C.E., Hernandez T.L., Kirwan J.P., Catalano P.M., Friedman J.E. (2007). Cellular Mechanisms for Insulin Resistance in Normal Pregnancy and Gestational Diabetes. Diabetes Care.

[9] Kampmann U., Knorr S., Fuglsang J., Ovesen P. (2019). Determinants of Maternal Insulin Resistance during Pregnancy: An Updated Overview. J. Diabetes Res..

[10] American Diabetes Association (2012). Diagnosis and Classification of Diabetes Mellitus. Diabetes Care.

[11] Rasmussen K.M., Yaktine A.L., Institute of Medicine (US) and National Research Council (US) Committee to Reexamine IOM Pregnancy Weight Guidelines (2009). Weight Gain During Pregnancy: Reexamining the Guidelines.

[12] Nowak C., Hetty S., Salihovic S., Castillejo-Lopez C., Ganna A., Cook N.L., Broeckling C.D., Prenni J.E., Shen X., Giedraitis V. (2018). Glucose challenge metabolomics implicates medium-chain acylcarnitines in insulin resistance. Sci. Rep..

[13] Miki T., Lee E.Y., Eguchi A., Sakurai K., Sawabe Y., Yoshida T., Saito K., Yokoh H., Ishikawa K., Yokote K. (2018). Accelerated oligosaccharide absorption and altered serum metabolites during oral glucose tolerance test in young Japanese with impaired glucose tolerance. J. Diabetes Investig..

[14] Bentley-Lewis R., Xiong G., Lee H., Yang A., Huynh J., Kim C. (2014). Metabolomic analysis reveals amino-acid responses to an oral glucose tolerance test in women with prior history of gestational diabetes mellitus. J. Clin. Transl. Endocrinol..

[15] Ho J.E., Larson M.G., Vasan R.S., Ghorbani A., Cheng S., Rhee E.P., Florez J.C., Clish C.B., Gerszten R.E., Wang T.J. (2013). Metabolite Profiles During Oral Glucose Challenge. Diabetes.

[16] Zhao X., Peter A., Fritsche J., Elcnerova M., Fritsche A., Häring H.-U., Schleicher E.D., Xu G., Lehmann R. (2009). Changes of the plasma metabolome during an oral glucose tolerance test: Is there more than glucose to look at?. Am. J. Physiol. Endocrinol. Metab..

[17] Wildberg C., Masuch A., Budde K., Kastenmüller G., Artati A., Rathmann W., Adamski J., Kocher T., Völzke H., Nauck M. (2019). Plasma Metabolomics to Identify and Stratify Patients With Impaired Glucose Tolerance. J. Clin. Endocrinol. Metab..

[18] Shaham O., Wei R., Wang T.J., Ricciardi C., Lewis G.D., Vasan R.S., Carr S.A., Thadhani R., Gerszten R.E., Mootha V.K. (2008). Metabolic profiling of the human response to a glucose challenge reveals distinct axes of insulin sensitivity. Mol. Syst. Biol..

[19] Gelaye B., Clish C.B., Denis M., Larrabure G., Tadesse M.G., Deik A., Pierce K., Bullock K., Dennis C., Enquobahrie D.A. (2019). Metabolomics signatures associated with an oral glucose challenge in pregnant women. Diabetes Metab..

[20] Scholtens D.M., Bain J.R., Reisetter A.C., Muehlbauer M.J., Nodzenski M., Stevens R.D., Ilkayeva O., Lowe L.P., Metzger B.E., Newgard C.B. (2016). Metabolic Networks and Metabolites Underlie Associations Between Maternal Glucose During Pregnancy and Newborn Size at Birth. Diabetes.

[21] Wang T.J., Ngo D., Psychogios N., Dejam A., Larson M.G., Vasan R.S., Ghorbani A., O’Sullivan J., Cheng S., Rhee E.P. (2013). 2-Aminoadipic acid is a biomarker for diabetes risk. J. Clin. Investig..

[22] Lehmann R., Friedrich T., Krebiehl G., Sonntag D., Häring H.-U., Fritsche A., Hennige A.M. (2015). Metabolic profiles during an oral glucose tolerance test in pregnant women with and without gestational diabetes. Exp. Clin. Endocrinol. Diabetes.

[23] Jovanovic L., Metzger B.E., Knopp R.H., Conley M.R., Park E., Lee Y.J., Simpson J.L., Holmes L., Aarons J.H., Mills J.L. (1998). The Diabetes in Early Pregnancy Study: Beta-hydroxybutyrate levels in type 1 diabetic pregnancy compared with normal pregnancy. NICHD-Diabetes in Early Pregnancy Study Group (DIEP). National Institute of Child Health and Development. Diabetes Care.

[24] Jovanovic L. (2004). Nutrition and pregnancy: The link between dietary intake and diabetes. Curr. Diabetes Rep..

[25] Nahavandi S., Seah J., Shub A., Houlihan C., Ekinci E.I. (2018). Biomarkers for Macrosomia Prediction in Pregnancies Affected by Diabetes. Front. Endocrinol..

[26] Ryckman K.K., Donovan B.M., Fleener D.K., Bedell B., Borowski K.S. (2016). Pregnancy-Related Changes of Amino Acid and Acylcarnitine Concentrations: The Impact of Obesity. AJP Rep..

[27] Schoderbeck M., Auer B., Legenstein E., Genger H., Sevelda P., Salzer H., Marz R., Lohninger A. (1995). Pregnancy-related changes of carnitine and acylcarnitine concentrations of plasma and erythrocytes. J. Perinat. Med..

[28] Wang G., Sun Q., Liang L., Clash C., Zhang C., Hong X., Ji Y., Radovick S., Pearson C., Bartell T.R. (2019). Inter-generational link of obesity in term and preterm births: Role of maternal plasma acylcarnitines. Int. J. Obes..

[29] Zhu Y., Li M., Rahman M.L., Hinkle S.N., Wu J., Weir N.L., Lin Y., Yang H., Tsai M.Y., Ferrara A. (2019). Plasma phospholipid n-3 and n-6 polyunsaturated fatty acids in relation to cardiometabolic markers and gestational diabetes: A longitudinal study within the prospective NICHD Fetal Growth Studies. PLoS Med..

[30] Pusl T., Beuers U. (2007). Intrahepatic cholestasis of pregnancy. Orphanet J. Rare Dis..

[31] Brites D. (2002). Intrahepatic cholestasis of pregnancy: Changes in maternal-fetal bile acid balance and improvement by ursodeoxycholic acid. Ann. Hepatol..

[32] Gottlieb A., Canbay A. (2019). Why Bile Acids Are So Important in Non-Alcoholic Fatty Liver Disease (NAFLD) Progression. Cells.

[33] Mantovani A., Byrne C.D., Bonora E., Targher G. (2018). Nonalcoholic Fatty Liver Disease and Risk of Incident Type 2 Diabetes: A Meta-analysis. Diabetes Care.

[34] Huynh J., Xiong G., Bentley-Lewis R. (2014). Systematic review A systematic review of metabolite profiling in gestational diabetes mellitus. Diabetologia.

[35] Trak-Fellermeier M.A., Campos M., Meléndez M., Pomeroy J., Palacios C., Rivera-Viñas J., Méndez K., Febo I., Willett W., Gillman M.W. (2019). PEARLS randomized lifestyle trial in pregnant Hispanic women with overweight/obesity: Gestational weight gain and offspring birthweight. Diabetes Metab. Syndr. Obes..

[36] Lagiou P., Tamimi R.M., Mucci L.A., Adami H.-O., Hsieh C.-C., Trichopoulos D. (2004). Diet during pregnancy in relation to maternal weight gain and birth size. Eur. J. Clin. Nutr..

[37] Olson C.M., Strawderman M.S., Reed R.G. (2004). Efficacy of an intervention to prevent excessive gestational weight gain. Am. J. Obstet. Gynecol..

[38] Stuebe A.M., Oken E., Gillman M.W. (2009). Associations of diet and physical activity during pregnancy with risk for excessive gestational weight gain. Am. J. Obstet. Gynecol..

[39] Izadi V., Tehrani H., Haghighatdoost F., Dehghan A., Surkan P.J., Azadbakht L. (2016). Adherence to the DASH and Mediterranean diets is associated with decreased risk for gestational diabetes mellitus. Nutrition.

[40] Assaf-Balut C., Garcia de la Torre N., Durán A., Fuentes M., Bordiú E., del Valle L., Valerio J., Familiar C., Jiménez I., Herraiz M.A. (2018). Medical nutrition therapy for gestational diabetes mellitus based on Mediterranean Diet principles: A subanalysis of the St Carlos GDM Prevention Study. BMJ Open Diabetes Res. Care.

[41] Torres R., Soltero S., Trak M.A., Tucker C.M., Mendez K., Campos M., Willett W., Melendez M., Gillman M.W., Franks P.W. (2016). Lifestyle modification intervention for overweight and obese Hispanic pregnant women: Development, implementation, lessons learned and future applications. Contemp. Clin. Trials Commun..

[42] Clifton R.G., Evans M., Cahill A.G., Franks P.W., Gallagher D., Phelan S., Pomeroy J., Redman L.M., Van Horn L. (2016). LIFE-Moms Research Group Design of lifestyle intervention trials to prevent excessive gestational weight gain in women with overweight or obesity. Obesity.

[43] Metzger B.E., Gabbe S.G., Persson B., Lowe L.P., Dyer A.R., Oats J.J.N., Buchanan T.A. (2010). International Association of Diabetes and Pregnancy Study Groups Recommendations on the Diagnosis and Classification of Hyperglycemia in Pregnancy: Response to Weinert. Diabetes Care.

[44] Tucker K.L., Maras J., Champagne C., Connell C., Goolsby S., Weber J., Zaghloul S., Carithers T., Bogle M.L. (2005). A regional food-frequency questionnaire for the US Mississippi Delta. Public Health Nutr..

[45] Zeng Z., Liu F., Li S. (2017). Metabolic Adaptations in Pregnancy: A Review. Ann. Nutr. Metab..

[46] Rodrigo N., Glastras S.J. (2018). The Emerging Role of Biomarkers in the Diagnosis of Gestational Diabetes Mellitus. J. Clin. Med..

