# Supplementary material for: Changes in Metabolites During an Oral Glucose Tolerance Test in Early and Mid-Pregnancy: Findings from the PEARLS Randomized, Controlled Lifestyle Trial
Source: Metabolites. 2020 Jul 10;10(7):284. doi: 10.3390/metabo10070284 (PMC7408149; doi:10.3390/metabo10070284)
Supplement: Supplementary file 1 [file metabolites-10-00284-s001.pdf]

Supplemental Material

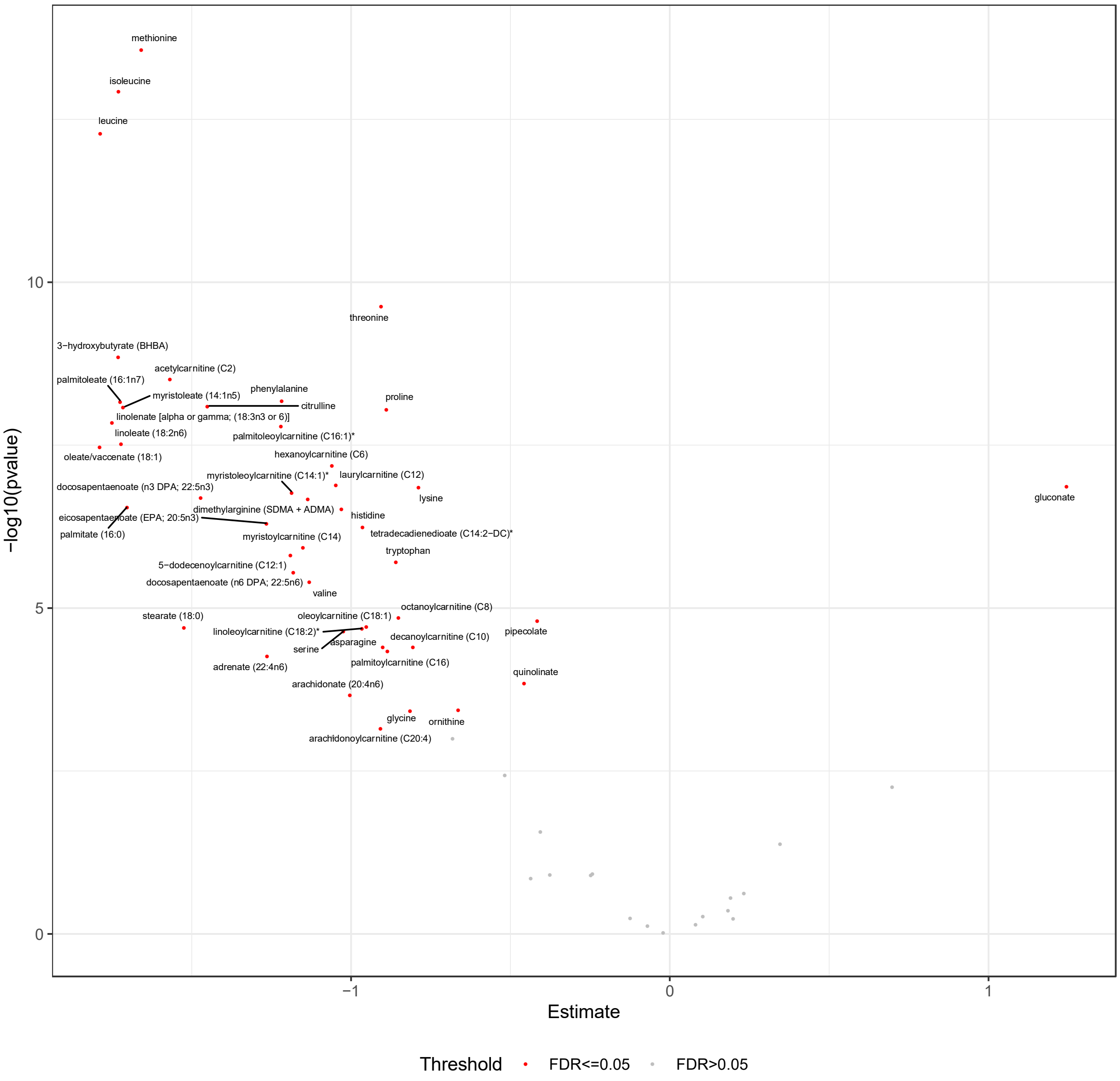

Figure S1. Mean Δfast-120min for metabolites during mid-pregnancy. This volcano plot displays mean Δfast-120min post-OGTT and $-\log_{10}$ *P* values for all candidate metabolites (n=65) among PEARLS participants at the mid-pregnancy visit (about 36 weeks) (n=18). Metabolites labeled with red passed the false discovery rate threshold of $p < 0.05$. Abbreviations: FDR, false discovery rate; OGTT, oral glucose tolerance test; PEARLS, Pregnancy and EARly Lifestyle improvement Study; Δfast-120min, changing in glucose from fasting to 120 minutes during the OGTT.

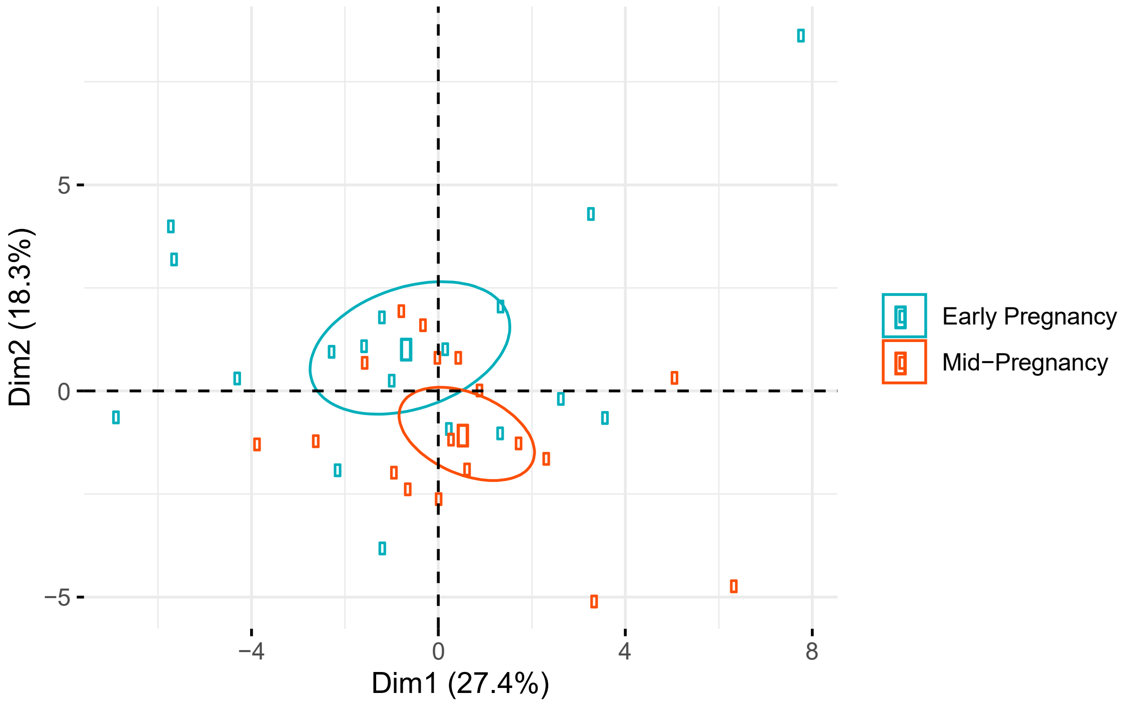

Figure S2. Score plot of principle component (PC) 1 versus PC2 for changes in metabolites from fasting to 120 minutes by pregnancy time. Comparison of changes in metabolites from fasting to 120 minutes (Δfast-120min) post-oral glucose tolerance test at early and mid-pregnancy for PEARLS participants (n=18). Depicts plot of first two components of each principal component (PC) analysis. Dimension 1 (Dim1) represents PC1 and Dimension 2 (Dim2) represents PC2. Percentage of variation in Δfast-120min explained by each PC is presented in parentheses. Ellipses represent 95% confidence intervals.

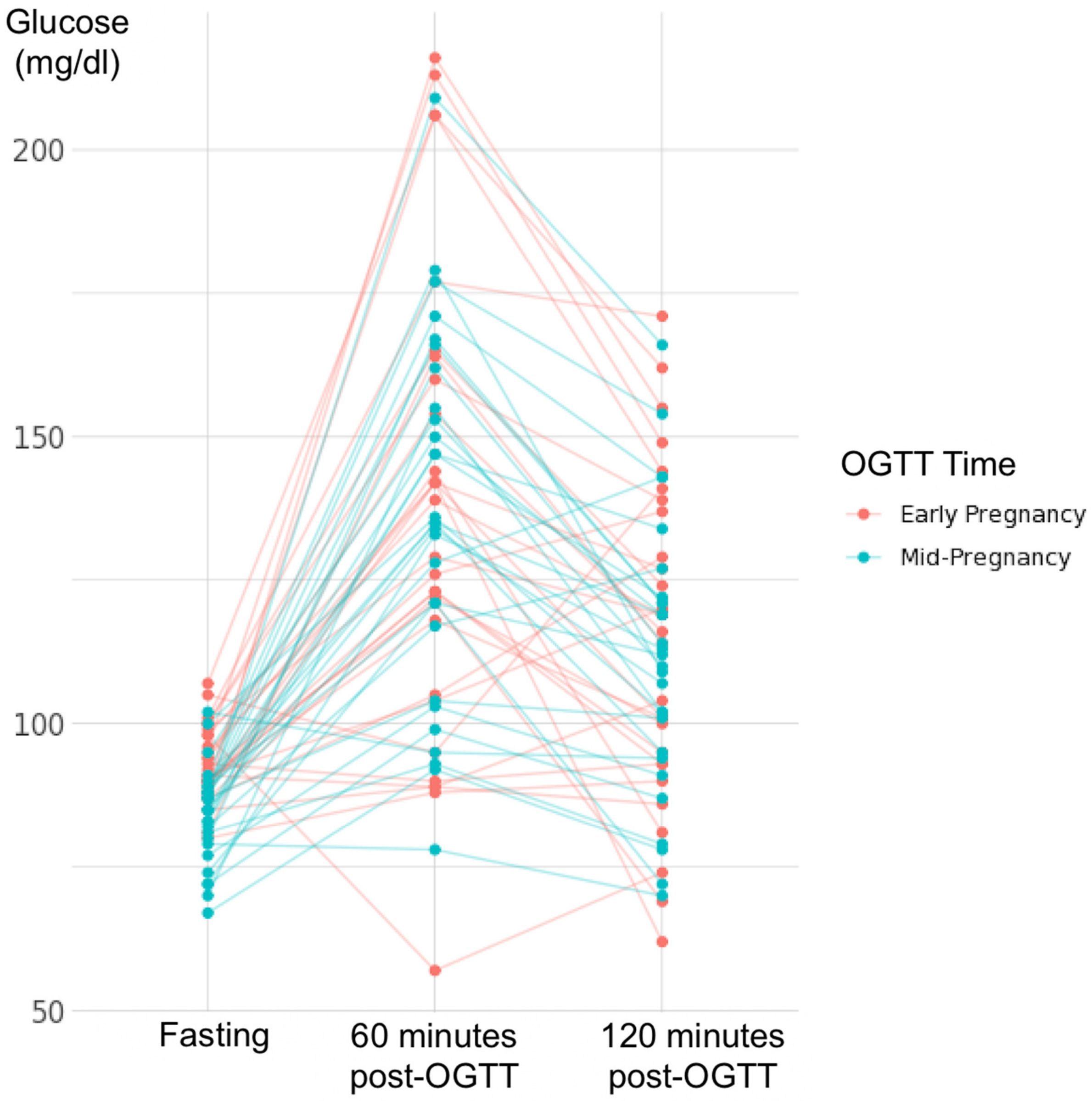

Figure S3. Changes in glucose during an oral glucose tolerance test (OGTT) at early and mid-pregnancy by participant in the Pregnancy and EARly Lifestyle improvement Study (PEARLS). *P*>0.05 for paired Student's t-test comparing mean change in glucose from fasting to 120 minutes post-OGTT conducted at early versus mid-pregnancy.

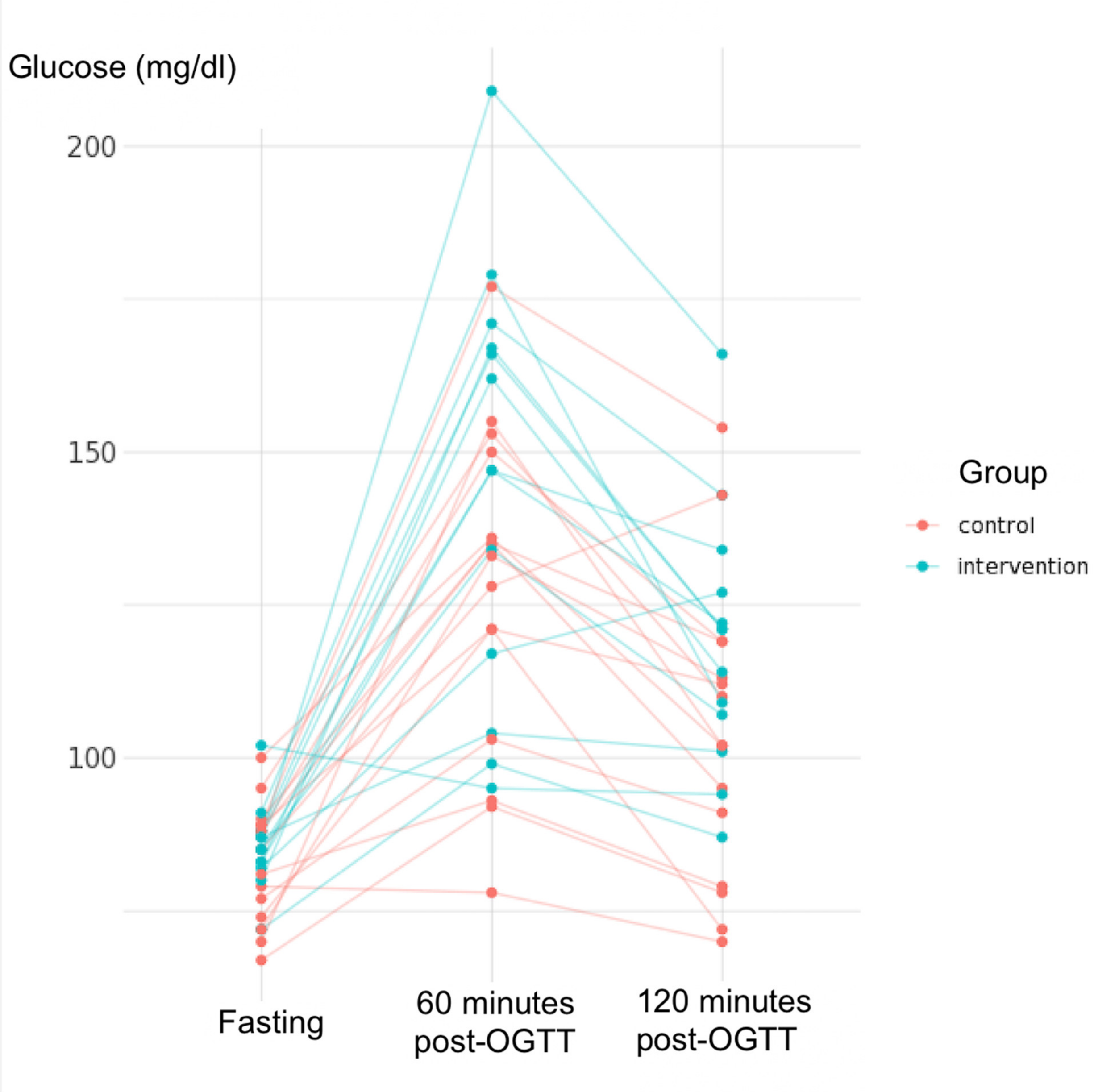

Figure S4. Changes in glucose during an oral glucose tolerance test (OGTT) by intervention status at mid-pregnancy and by participants of the Pregnancy and EARly Lifestyle improvement Study (PEARLS). *P*>0.05 for Student's t-test comparing mean change in glucose from fasting to 120 minutes post-OGTT among the intervention and control groups at mid-pregnancy.

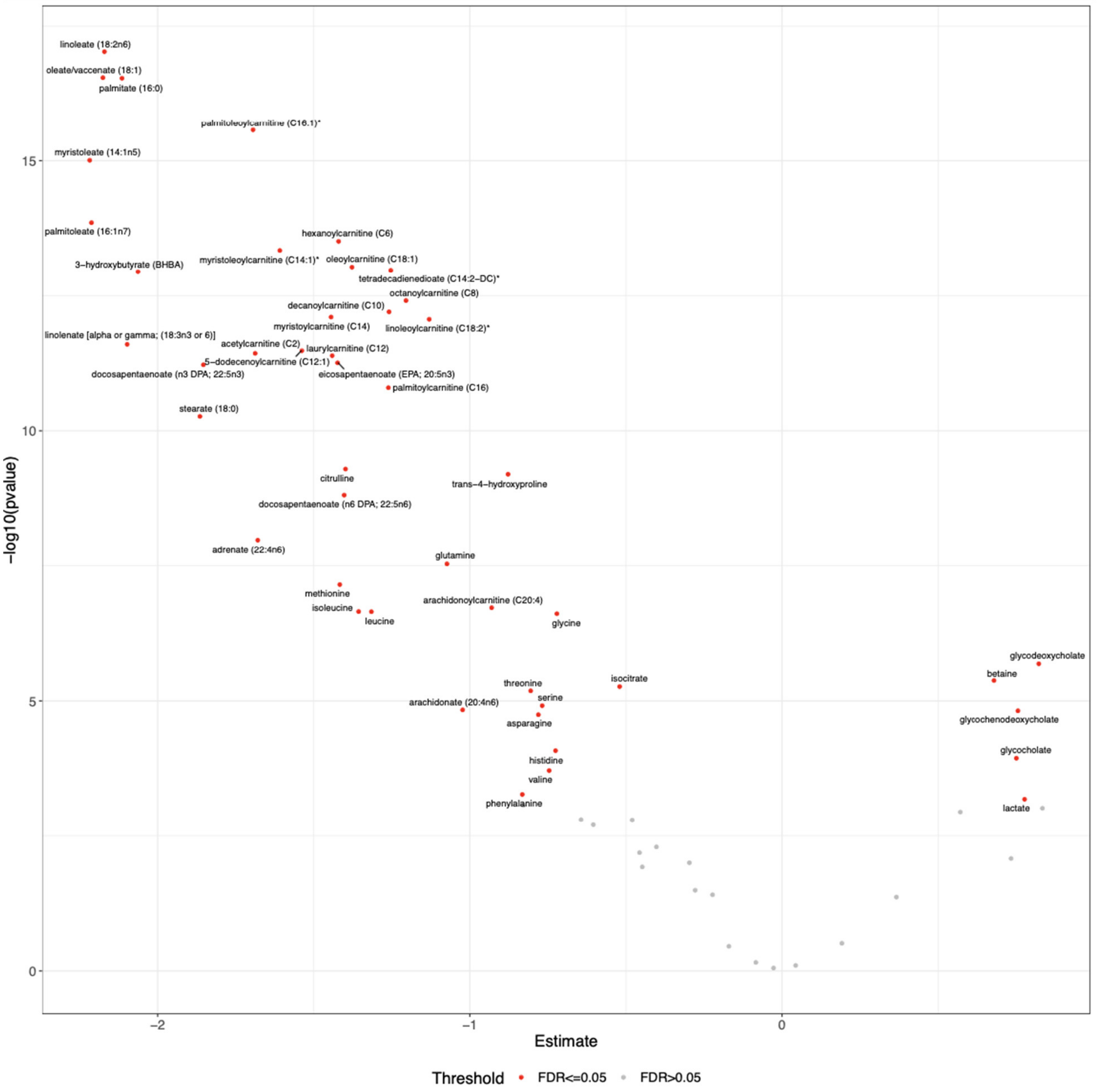

Figure S5. Mean Δfast-120min for metabolites at early pregnancy adjusting for maternal age. This volcano plot displays mean Δfast-120min post-OGTT and -log10 P values for all candidate metabolites (n=65) among PEARLS participants at the baseline visit (n=29). Metabolites labeled with red passed the false discovery rate threshold of $p < 0.05$. Abbreviations: FDR, false discovery rate; OGTT, oral glucose tolerance test; PEARLS, Pregnancy and EARly Lifestyle improvement Study; Δfast-120min, changing in glucose from fasting to 120 minutes during the OGTT.

Table S1. Mean difference in top principal component (PC) scores for fasting metabolites at early (n=29) and mid-pregnancy (n=18) in intervention group compared to the control group among PEARLS participants.

	β (SE)	*P* value
**Baseline**		
PC1	0.9 (1.5)	0.58
PC2	1.1 (1.2)	0.37
**Mid-Pregnancy**		
PC1	-2.7 (1.6)	0.12
PC2	-1.5 (1.2)	0.23

Table S2. Factor loadings for linear combination of metabolites defining principal component 1 (PC1) for fasting metabolites at mid-pregnancy among Pregnancy and EARly Lifestyle improvement Study (PEARLS) participants.

Metabolite	PC1 Weight at mid-pregnancy[1]
**myristoleoylcarnitine (C14:1)***	**-0.24**
**palmitoleoylcarnitine (C16:1)***	**-0.24**
**leucine**	0.00
**linoleoylcarnitine (C18:2)***	-0.15
**5-dodecenoylcarnitine (C12:1)**	**-0.24**
**myristoylcarnitine (C14)**	**-0.20**
**palmitoylcarnitine (C16)**	**-0.21**
**3-hydroxybutyrate (BHBA)**	**-0.21**
**oleoylcarnitine (C18:1)**	0.06
**asparagine**	-0.13
**glycodeoxycholate**	0.14
**tryptophan**	0.06
**glutamine**	-0.05
**adrenate (22:4n6)**	0.02
**isoleucine**	-0.15
**linoleate (18:2n6)**	-0.16
**ornithine**	0.09
**glycocholate**	0.09
**linolenate [alpha or gamma; (18:3n3 or 6)]**	-0.12
**pyruvate**	0.10
**hexanoylcarnitine (C6)**	**-0.22**
**oleate/vaccenate (18:1)**	-0.18
**acetylcarnitine (C2)**	-0.12
**glycochenodeoxycholate**	0.08
**palmitoleate (16:1n7)**	-0.14
**laurylcarnitine (C12)**	**-0.22**

**tetradecadienedioate (C14:2-DC)***	-0.11
**myristoleate (14:1n5)**	-0.09
**valine**	0.00
**methionine**	-0.03
**lactate**	**-0.20**
**decanoylcarnitine (C10)**	0.03
**dimethylarginine (SDMA + ADMA)**	-0.09
**octanoylcarnitine (C8)**	**-0.20**
**alanine**	0.04
**arginine**	0.04
**phenylalanine**	-0.02
**docosapentaenoate (n6 DPA; 22:5n6)**	-0.09
**isocitrate**	-0.08
**threonine**	-0.02
**eicosapentaenoate (EPA; 20:5n3)**	-0.14
**lysine**	-0.07
**uridine**	-0.05
**palmitate (16:0)**	-0.14
**glycine**	0.07
**stearoylcarnitine (C18)**	-0.13
**betaine**	-0.17
**stearate (18:0)**	-0.12
**2-aminoadipate**	-0.01
**aspartate**	0.06
**quinolinate**	-0.06
**histidine**	0.04
**docosapentaenoate (n3 DPA; 22:5n3)**	-0.18
**citrulline**	0.07
**urate**	-0.15
**fructose**	0.01
**tyrosine**	-0.01
**proline**	0.00
**hypoxanthine**	0.03
**arachidonate (20:4n6)**	-0.13
**taurine**	0.06
**trans-4-hydroxyproline**	0.10
**glutamate**	0.11
**serine**	0.07
**arachidonoylcarnitine (C20:4)**	-0.12

[1]Weights correspond to PCA presented in Figure 2B.

Table S3. Mean change in metabolites from fasting to 120 minutes post-OGTT (Δfast-120min) for targeted metabolites during early versus mid-pregnancy among PEARLS participants.

Metabolite	HMDB ID	Biological Description	Early Pregnancy[1]		Mid-Pregnancy[2]		Early vs. Mid-Pregnancy[3]
			Mean (SD) Change	FDR-Adjusted *P*-value	Mean (SD) Change	FDR-Adjusted *P*-value	*P*-value
**myristoleate (14:1n5)**	HMDB02000	Long Chain Monounsaturated Fatty Acid	-2.2 (0.7)	**6.35E-14**	-1.5 (0.7)	**1.37E-06**	0.001
**tetradecadienedioate (C14:2-DC)***	NA	Fatty Acid, Dicarboxylate	-1.3 (0.5)	**6.95E-12**	-0.8 (0.4)	**5.55E-06**	0.008
**trans-4-hydroxyproline**	HMDB00725	Urea cycle; Arginine and Proline Metabolism	-0.8 (0.3)	**4.11E-08**	-0.4 (0.5)	0.25	0.008
**glycocholate**	HMDB00138	Primary Bile Acid Metabolism	0.7 (0.7)	**7.44E-03**	0.1 (0.7)	0.99	0.01
**palmitoleate (16:1n7)**	HMDB03229	Long Chain Monounsaturated Fatty Acid	-2.2 (0.9)	**9.09E-13**	-1.6 (0.6)	**2.09E-07**	0.02
**laurylcarnitine (C12)**	HMDB02250	Fatty Acid Metabolism (Acyl Carnitine, Medium Chain)	-1.5 (0.7)	**2.66E-10**	-1.0 (0.5)	**6.21E-05**	0.02
**dimethylarginine (SDMA + ADMA)**	HMDB01539	Urea cycle; Arginine and Proline Metabolism	-0.5 (0.8)	0.10	-1.0 (0.5)	**2.29E-06**	0.02
**octanoylcarnitine (C8)**	HMDB00791	Fatty Acid Metabolism (Acyl Carnitine, Medium Chain)	-1.3 (0.4)	**2.54E-11**	-0.9 (0.5)	**1.98E-04**	0.02
**decanoylcarnitine (C10)**	HMDB00651	Fatty Acid Metabolism (Acyl Carnitine, Medium Chain)	-1.3 (0.5)	**4.13E-11**	-0.9 (0.6)	**1.15E-03**	0.02
**docosapentaenoate (n3 DPA; 22:5n3)**	HMDB06528	Long Chain Polyunsaturated Fatty Acid (n3 and n6)	-2.0 (0.8)	**3.92E-10**	-1.5 (0.7)	**1.35E-05**	0.02
**oleate/vaccenate (18:1)**	NA	Long Chain Monounsaturated Fatty Acid	-2.3 (0.8)	**1.81E-10**	-1.6 (0.8)	**6.06E-06**	0.02
**linolenate [alpha or gamma; (18:3n3 or 6)]**	HMDB03073	Long Chain Polyunsaturated Fatty Acid (n3 and n6)	-2.2 (0.8)	**1.64E-10**	-1.6 (0.7)	**9.81E-07**	0.02
**betaine**	HMDB00043	Glycine, Serine and Threonine Metabolism	0.7 (0.7)	**2.72E-04**	0.2 (0.5)	0.99	0.03
**linoleate (18:2n6)**	HMDB00673	Long Chain Polyunsaturated Fatty Acid (n3 and n6)	-2.3 (0.9)	**4.60E-10**	-1.6 (0.7)	**2.13E-06**	0.03
**docosapentaenoate (n6 DPA; 22:5n6)**	HMDB01976	Long Chain Polyunsaturated Fatty Acid (n3 and n6)	-1.6 (0.7)	**9.94E-08**	-1.1 (0.7)	**1.67E-04**	0.03
**3-hydroxybutyrate (BHBA)**	HMDB00357	Ketone Bodies	-2.1 (0.6)	**7.39E-12**	-1.6 (0.7)	**8.92E-05**	0.03
**isocitrate**	HMDB00193	Tricarboxylic Acid Cycle	-0.6 (0.5)	**3.51E-04**	0.02 (0.9)	0.99	0.03
**palmitate (16:0)**	HMDB00220	Long Chain Saturated Fatty Acid	-2.3 (0.9)	**1.81E-10**	-1.6 (0.8)	**1.30E-05**	0.03
**glycochenodeoxycholate**	HMDB00637	Primary Bile Acid Metabolism	0.8 (0.8)	**9.84E-04**	0.3 (0.7)	0.99	0.03
**hexanoylcarnitine (C6)**	HMDB00705	Fatty Acid Metabolism (Acyl Carnitine, Medium Chain)	-1.5 (0.5)	**2.01E-12**	-1.2 (0.6)	**5.27E-06**	0.04
**myristoleoylcarnitine (C14:1)***	NA	Fatty Acid Metabolism (Acyl Carnitine, Monounsaturated)	-1.6 (0.6)	**2.98E-12**	-1.2 (0.6)	**8.61E-06**	0.05
**adrenate (22:4n6)**	HMDB02226	Long Chain Polyunsaturated Fatty Acid (n3 and n6)	-1.8 (1.0)	**6.80E-07**	-1.2 (1.0)	**4.75E-03**	0.07

**5-dodecenoylcarnitine (C12:1)**	HMDB13326	Fatty Acid Metabolism (Acyl Carnitine, Monounsaturated)	-1.6 (0.7)	**2.15E-10**	-1.2 (0.7)	**2.06E-04**	0.08
**stearate (18:0)**	HMDB00827	Long Chain Saturated Fatty Acid	-2.0 (0.8)	**3.52E-09**	-1.5 (1.0)	**9.35E-04**	0.09
**hypoxanthine**	HMDB00157	Purine Metabolism, (Hypo)Xanthine/Inosine containing	0.8 (1.6)	0.16	0.1 (0.9)	0.99	0.09
**glycodeoxycholate**	HMDB00631	Secondary Bile Acid Metabolism	0.7 (0.8)	**1.33E-04**	0.2 (0.7)	0.99	0.10
**palmitoleoylcarnitine (C16:1)***	NA	Fatty Acid Metabolism (Acyl Carnitine, Monounsaturated)	-1.5 (0.4)	**1.73E-14**	-1.3 (0.6)	**5.90E-04**	0.14
**lysine**	HMDB00182	Lysine Metabolism	-0.5 (0.6)	0.33	-0.7 (0.3)	**6.25E-06**	0.15
**acetylcarnitine (C2)**	HMDB00201	Fatty Acid Metabolism (Acyl Carnitine, Short Chain)	-1.6 (0.6)	**2.41E-10**	-1.3 (0.5)	**6.53E-08**	0.16
**oleoylcarnitine (C18:1)**	HMDB05065	Fatty Acid Metabolism (Acyl Carnitine, Monounsaturated)	-1.3 (0.6)	**6.08E-12**	-1.0 (0.7)	**1.71E-03**	0.20
**pyruvate**	HMDB00243	Glycolysis, Gluconeogenesis, and Pyruvate Metabolism	0.6 (1.0)	0.07	0.3 (0.6)	0.99	0.27
**palmitoylcarnitine (C16)**	HMDB00222	Fatty Acid Metabolism (Acyl Carnitine, Long Chain Saturated)	-1.2 (0.7)	**1.04E-09**	-0.9 (0.7)	**3.99E-03**	0.29
**ornithine**	HMDB03374	Urea cycle; Arginine and Proline Metabolism	-0.4 (0.6)	0.99	-0.5 (0.5)	**0.02**	0.32
**taurine**	HMDB00251	Methionine, Cysteine, SAM and Taurine Metabolism	0.3 (1.0)	0.99	-0.01 (1.1)	0.99	0.34
**myristoylcarnitine (C14)**	HMDB05066	Fatty Acid Metabolism (Acyl Carnitine, Long Chain Saturated)	-1.4 (0.6)	**5.15E-11**	-1.2 (0.7)	**9.28E-05**	0.34
**glutamine**	HMDB00641	Glutamate Metabolism	-1.1 (0.7)	**1.89E-06**	-0.9 (0.6)	**2.48E-06**	0.35
**linoleoylcarnitine (C18:2)***	HMDB06469	Fatty Acid Metabolism (Acyl Carnitine, Polyunsaturated)	-1.1 (0.5)	**5.63E-11**	-0.9 (0.7)	**1.35E-03**	0.37
**proline**	HMDB00162	Urea cycle; Arginine and Proline Metabolism	-0.6 (0.7)	0.77	-0.7 (0.3)	**9.73E-08**	0.44
**aspartate**	HMDB00191	Alanine and Aspartate Metabolism	0.1 (1.1)	0.99	-0.2 (0.8)	0.99	0.45
**serine**	HMDB00187	Glycine, Serine and Threonine Metabolism	-0.8 (0.8)	**7.93E-04**	-1.0 (0.7)	**2.15E-03**	0.50
**arachidonate (20:4n6)**	HMDB01043	Long Chain Polyunsaturated Fatty Acid (n3 and n6)	-1.1 (0.9)	**9.48E-04**	-1.0 (0.9)	**0.02**	0.51
**glutamate**	HMDB00148	Glutamate Metabolism	-0.1 (1.1)	0.99	-0.3 (0.8)	0.99	0.52
**phenylalanine**	HMDB00159	Phenylalanine Metabolism	-1.0 (1.0)	**0.03**	-1.2 (0.6)	**5.64E-06**	0.54
**eicosapentaenoate (EPA; 20:5n3)**	HMDB01999	Long Chain Polyunsaturated Fatty Acid (n3 and n6)	-1.5 (0.6)	**3.60E-10**	-1.3 (0.7)	**3.95E-05**	0.55
**quinolinate**	HMDB00232	Nicotinate and Nicotinamide Metabolism	-0.3 (0.6)	0.99	-0.4 (0.4)	0.05	0.57
**tryptophan**	HMDB00929	Tryptophan Metabolism	-0.7 (0.8)	0.42	-0.7 (0.4)	**8.14E-07**	0.60
**stearoylcarnitine (C18)**	HMDB00848	Fatty Acid Metabolism (Acyl Carnitine, Long Chain Saturated)	-0.6 (1.1)	0.10	-0.4 (0.7)	0.99	0.66
**glycine**	HMDB00123	Glycine, Serine and Threonine Metabolism	-0.7 (0.6)	**1.58E-05**	-0.8 (0.7)	**0.01**	0.67
**lactate**	HMDB00190	Glycolysis, Gluconeogenesis, and Pyruvate Metabolism	0.7 (1.3)	**0.04**	0.6 (0.9)	0.83	0.68
**threonine**	HMDB00167	Glycine, Serine and Threonine Metabolism	-0.9 (0.6)	**4.21E-04**	-0.8 (0.2)	**2.06E-09**	0.70

**urate**	HMDB00289	Purine Metabolism, (Hypo)Xanthine/Inosine containing	-0.3 (0.5)	0.65	-0.2 (0.6)	0.99	0.70
**isoleucine**	HMDB00172	Leucine, Isoleucine and Valine Metabolism	-1.5 (0.6)	**1.44E-05**	-1.6 (0.3)	**1.33E-11**	0.73
**fructose**	HMDB00660	Fructose, Mannose and Galactose Metabolism	0.5 (1.6)	0.54	0.7 (0.9)	0.43	0.73
**leucine**	NA	Leucine, Isoleucine and Valine Metabolism	-1.5 (0.7)	**1.45E-05**	-1.5 (0.4)	**1.36E-10**	0.73
**histidine**	HMDB00177	Histidine Metabolism	-0.9 (0.7)	**5.40E-03**	-1.0 (0.5)	**1.66E-05**	0.81
**valine**	HMDB00883	Leucine, Isoleucine and Valine Metabolism	-0.9 (0.7)	**0.01**	-0.9 (0.5)	**1.20E-04**	0.85
**alanine**	HMDB00161	Alanine and Aspartate Metabolism	-0.1 (0.8)	0.99	-0.1 (0.5)	0.99	0.87
**arachidonoylcarnitine (C20:4)**	NA	Fatty Acid Metabolism (Acyl Carnitine, Polyunsaturated)	-0.8 (0.7)	**1.22E-05**	-0.7 (0.7)	**0.03**	0.89
**methionine**	HMDB00696	Methionine, Cysteine, SAM and Taurine Metabolism	-1.6 (0.8)	**4.57E-06**	-1.5 (0.4)	**2.07E-10**	0.90
**2-aminoadipate**	HMDB00510	Lysine Metabolism	-0.3 (1.0)	0.99	-0.4 (0.9)	0.99	0.91
**arginine**	HMDB00517	Urea cycle; Arginine and Proline Metabolism	-0.6 (1.0)	**0.01**	-0.7 (0.7)	0.05	0.92
**uridine**	HMDB00296	Pyrimidine Metabolism, Uracil containing	0.3 (0.7)	0.99	0.2 (0.6)	0.99	0.92
**asparagine**	HMDB00168	Alanine and Aspartate Metabolism	-0.9 (0.7)	**1.16E-03**	-0.9 (0.7)	**1.68E-03**	0.93
**citrulline**	HMDB00094	Urea cycle; Arginine and Proline Metabolism	-1.5 (0.6)	**3.30E-08**	-1.5 (0.6)	**3.31E-07**	0.97
**tyrosine**	HMDB00158	Tyrosine Metabolism	-1.0 (1.0)	0.06	-1.0 (1.0)	**0.04**	0.99

[1] Values represent mean changes in metabolites at fasting versus 120 min at the OGTT conducted at during early pregnancy (N=29), and *p*-values represent paired Student *t* tests to examine difference between these two time points after FDR correction for multiple testing. [2] Values represent mean changes in metabolites at fasting versus 120 min at the OGTT conducted at mid-pregnancy (about 36 weeks) (N=18), and *p*-values represent paired Student *t* tests to examine difference between these two time points after FDR correction for multiple testing. [3] P-values represent paired Student's *t* tests without FDR correction comparing mean differences during the OGTT (fasting to 120 minutes) at early versus mid-pregnancy (N=18). Abbreviations: FDR, False Discovery Rate; OGTT, oral glucose tolerance test; PEARLS, Pregnancy and EARly Lifestyle improvement Study; SD, standard deviation.

Table S4. Mean difference in top principal component (PC) scores for changes in metabolites from fasting to 120 minutes (Δfast-120min) during early compared to mid-pregnancy (n=18).

	**Percent of variation in Δfast-120min explained by PC**	**β (SE)**	***P* value**
PC1	27.4%	1.2 (1.1)	0.26
PC2	18.3%	-2.1 (0.8)	**0.01**

Table S5. Factor loadings from principal component (PC) analysis at early and mid-pregnancy. Loadings are derived from PCA comparing metabolite levels during an oral glucose tolerance test (OGTT) among Pregnancy and EARly Lifestyle improvement Study (PEARLS) participants during early (n=29) and mid- (n=18) pregnancy, and PC analysis comparing changes in metabolites from fasting to 120 minutes (Δfast-120min) at baseline versus 36 weeks.

Metabolite	HMDB ID	References[1]	Biological Pathway	PC1: OGTT Metabolites Early Pregnancy[2]	PC1: OGTT Metabolites Mid-Pregnancy[3]	PC2: ΔFast-120Min Early versus Mid-Pregnancy[4]
**oleate/vaccenate (18:1)**	NA	(1–3)	Long Chain Monounsaturated Fatty Acid	0.21	0.18	-0.17
**palmitoleate (16:1n7)**	HMDB03229	(1,2)	Long Chain Monounsaturated Fatty Acid	0.20	0.18	-0.10
**linoleate (18:2n6)**	HMDB00673	(1,3–5)	Long Chain Polyunsaturated Fatty Acid (n3 and n6)	0.20	0.19	0.08
**palmitate (16:0)**	HMDB00220	(2,4,5)	Long Chain Saturated Fatty Acid	0.20	0.19	0.13
**linolenate [alpha or gamma; (18:3n3 or 6)]**	HMDB03073	(4,5)	Long Chain Polyunsaturated Fatty Acid (n3 and n6)	0.20	0.18	0.00
**myristoleate (14:1n5)**	HMDB02000	(1,2,4–6)	Long Chain Monounsaturated Fatty Acid	0.20	0.16	0.09
**docosapentaenoate (n3 DPA; 22:5n3)**	HMDB06528	(4,5)	Long Chain Polyunsaturated Fatty Acid (n3 and n6)	0.19	0.19	0.03
**3-hydroxybutyrate (BHBA)**	HMDB00929	(6–8)	Ketone Bodies	0.19	0.15	0.12
**palmitoleoylcarnitine (C16:1)***	HMDB00641	(6,7)	Fatty Acid Metabolism (Acyl Carnitine, Monounsaturated)	0.18	0.19	0.15
**adrenate (22:4n6)**	HMDB02226	(3,5)	Long Chain Polyunsaturated Fatty Acid (n3 and n6)	0.18	0.17	-0.17
**stearate (18:0)**	HMDB00827	(2,4)	Long Chain Saturated Fatty Acid	0.18	0.16	-0.20
**myristoleoylcarnitine (C14:1)***	NA	(2,4–6)	Fatty Acid Metabolism (Acyl Carnitine, Monounsaturated)	0.17	0.19	0.11
**acetylcarnitine (C2)**	HMDB00201	(2,4–6)	Fatty Acid Metabolism (Acyl Carnitine, Short Chain)	0.17	0.15	0.08
**5-dodecenoylcarnitine (C12:1)**	HMDB13326	(2,4–6)	Fatty Acid Metabolism (Acyl Carnitine, Monounsaturated)	0.17	0.19	0.18
**myristoylcarnitine (C14)**	HMDB05066	(2,4–6)	Fatty Acid Metabolism (Acyl Carnitine, Long Chain Saturated)	0.17	0.19	0.10
**laurylcarnitine (C12)**	HMDB02250	(1,4,6)	Fatty Acid Metabolism (Acyl Carnitine, Medium Chain)	0.17	0.17	0.16
**leucine**	NA	(6–9)	Leucine, Isoleucine and Valine Metabolism	0.16	0.14	-0.10
**docosapentaenoate (n6 DPA; 22:5n6)**	HMDB01976	(4,5)	Long Chain Polyunsaturated Fatty Acid (n3 and n6)	0.16	0.14	0.21
**oleoylcarnitine (C18:1)**	HMDB05065	(2,4–6)	Fatty Acid Metabolism (Acyl Carnitine, Monounsaturated)	0.15	0.17	0.21
**hexanoylcarnitine (C6)**	HMDB00705	(2,5,6)	Fatty Acid Metabolism (Acyl Carnitine, Medium Chain)	0.15	0.19	0.13

**eicosapentaenoate (EPA; 20:5n3)**	HMDB01999	(3,5)	Long Chain Polyunsaturated Fatty Acid (n3 and n6)	0.15	0.17	0.08
**decanoylcarnitine (C10)**	HMDB00651	(1,2,4,6)	Fatty Acid Metabolism (Acyl Carnitine, Medium Chain)	0.15	0.17	0.11
**isoleucine**	HMDB00172	(4,6–9)	Leucine, Isoleucine and Valine Metabolism	0.15	0.13	0.04
**palmitoylcarnitine (C16)**	HMDB00222	(2,4–6)	Fatty Acid Metabolism (Acyl Carnitine, Long Chain Saturated)	0.14	0.17	0.12
**octanoylcarnitine (C8)**	HMDB00791	(2,4,6)	Fatty Acid Metabolism (Acyl Carnitine, Medium Chain)	0.14	0.17	0.18
**arachidonate (20:4n6)**	HMDB01043	(5,10)	Long Chain Polyunsaturated Fatty Acid (n3 and n6)	0.14	0.15	0.01
**methionine**	HMDB00696	(4,6–8)	Methionine, Cysteine, SAM and Taurine Metabolism	0.14	0.15	-0.01
**phenylalanine**	HMDB00159	(4,6–8)	Phenylalanine Metabolism	0.14	0.14	0.02
**tyrosine**	HMDB00158	(4,6,8)	Tyrosine Metabolism	0.12	0.1	0.21
**linoleoylcarnitine (C18:2)***	HMDB06469	(2,4–6)	Fatty Acid Metabolism (Acyl Carnitine, Polyunsaturated)	0.11	0.14	-0.15
**arachidonoylcarnitine (C20:4)**	NA	(2,4–6)	Fatty Acid Metabolism (Acyl Carnitine, Polyunsaturated)	0.11	0.12	-0.17
**serine**	HMDB00187	(4,6,7)	Glycine, Serine and Threonine Metabolism	0.11	0.06	-0.19
**valine**	HMDB00883	(4,6–8)	Leucine, Isoleucine and Valine Metabolism	0.10	0.08	-0.16
**citrulline**	HMDB00094	(3,4,6,8)	Urea cycle; Arginine and Proline Metabolism	0.10	0.09	0.01
**arginine**	HMDB00517	(4,6)	Urea cycle; Arginine and Proline Metabolism	0.10	0.04	-0.01
**tetradecadienedioate (C14:2-DC)***	NA	(2,4–6)	Fatty Acid, Dicarboxylate	0.09	0.10	0.01
**tryptophan**	HMDB00357	(4,6)	Tryptophan Metabolism	0.09	0.07	-0.13
**glutamine**	NA	(2,4–6)	Glutamate Metabolism	0.09	0.11	0.08
**dimethylarginine (SDMA + ADMA)**	HMDB01539	(1,4,6)	Urea cycle; Arginine and Proline Metabolism	0.09	0.12	-0.03
**lysine**	HMDB00182	(6–8)	Lysine Metabolism	0.08	0.09	0.01
**proline**	HMDB00162	(6,7)	Urea cycle; Arginine and Proline Metabolism	0.08	0.07	0.15
**histidine**	HMDB00177	(6–8)	Histidine Metabolism	0.08	0.09	-0.17
**glutamate**	HMDB00148	(4,6,7)	Glutamate Metabolism	0.08	-0.01	0.10
**stearoylcarnitine (C18)**	HMDB00848	(2,4–6)	Fatty Acid Metabolism (Acyl Carnitine, Long Chain Saturated)	0.07	0.12	0.03
**asparagine**	HMDB00168	(3,4,6,7)	Alanine and Aspartate Metabolism	0.07	0.09	0.04
**ornithine**	HMDB03374	(3,4,6,8)	Urea cycle; Arginine and Proline Metabolism	0.07	0.03	-0.07
**glycine**	HMDB00123	(3,4,6,7)	Glycine, Serine and Threonine Metabolism	0.07	0.06	0.21
**threonine**	HMDB00167	(6,7)	Glycine, Serine and Threonine Metabolism	0.06	0.08	0.12
**trans-4-hydroxyproline**	HMDB00725	(6,7)	Urea cycle; Arginine and Proline Metabolism	0.06	0.01	0.14
**aspartate**	HMDB00191	(4,6,7)	Alanine and Aspartate Metabolism	0.05	0.03	-0.19
**quinolinate**	HMDB00232	(6,9)	Nicotinate and Nicotinamide Metabolism	0.05	0.07	0.15
**uridine**	HMDB00296	(6,9)	Pyrimidine Metabolism, Uracil containing	0.04	0.02	0.03

**2-aminoadipate**	HMDB00510	(6,11)	Lysine Metabolism	0.04	0.05	-0.05
**urate**	HMDB00289	(1,6)	Purine Metabolism, (Hypo)Xanthine/Inosine containing	0.04	0.07	0.18
**alanine**	HMDB00161	(3,6,7)	Alanine and Aspartate Metabolism	0.03	0.03	-0.07
**taurine**	HMDB00251	(6,9)	Methionine, Cysteine, SAM and Taurine Metabolism	0.02	0.01	0.11
**isocitrate**	HMDB00193	(4,6)	TCA Cycle	0.01	0.02	0.12
**fructose**	HMDB00660	(4,6)	Fructose, Mannose and Galactose Metabolism	0.01	-0.06	0.15
**glycocholate**	HMDB00138	(2,6,8)	Primary Bile Acid Metabolism	-0.01	-0.03	0.13
**betaine**	HMDB00043	(3,6)	Glycine, Serine and Threonine Metabolism	-0.02	0.03	0.11
**hypoxanthine**	HMDB00157	(6,8)	Purine Metabolism, (Hypo)Xanthine/Inosine containing	-0.03	0.00	-0.02
**lactate**	HMDB00190	(3,4,6,8)	Glycolysis, Gluconeogenesis, and Pyruvate Metabolism	-0.03	-0.04	-0.04
**glycochenodeoxycholate**	HMDB00637	(2,5,6,8)	Primary Bile Acid Metabolism	-0.04	-0.05	-0.07
**glycodeoxycholate**	HMDB00631	(1,2,6)	Secondary Bile Acid Metabolism	-0.04	-0.07	-0.11
**pyruvate**	HMDB00243	(3,4,8)	Glycolysis, Gluconeogenesis, and Pyruvate Metabolism	-0.05	-0.08	0.17

[1] Previous studies that have observed significant changes in metabolite or related metabolites during an oral glucose tolerance test. [2] Factor loadings correspond to PC1 in Figure 3A. [3] Factor loadings correspond to PC1 in Figure 3B. [4] Factor loadings correspond to PC2 in Figure S2. We present loadings for only PC2 here because PC1 was significantly associated with the time during pregnancy that the OGTT was conducted.

References

1. Nowak C, Hetty S, Salihovic S, Castillejo-Lopez C, Ganna A, Cook NL, et al. Glucose challenge metabolomics implicates medium-chain acylcarnitines in insulin resistance. Sci Rep [Internet]. 2018 Jun 6 [cited 2019 Sep 23];8. Available from: https://www.ncbi.nlm.nih.gov/pmc/articles/PMC5989236/
2. Zhao X, Peter A, Fritsche J, Elcnerova M, Fritsche A, Häring H-U, et al. Changes of the plasma metabolome during an oral glucose tolerance test: is there more than glucose to look at? Am J Physiol Endocrinol Metab. 2009 Feb;296(2):E384-393.
3. Wildberg C, Masuch A, Budde K, Kastenmüller G, Artati A, Rathmann W, et al. Plasma Metabolomics to Identify and Stratify Patients With Impaired Glucose Tolerance. J Clin Endocrinol Metab. 2019 Dec 1;104(12):6357–70.
4. Scholtens DM, Bain JR, Reisetter AC, Muehlbauer MJ, Nodzenski M, Stevens RD, et al. Metabolic Networks and Metabolites Underlie Associations Between Maternal Glucose During Pregnancy and Newborn Size at Birth. Diabetes. 2016 Jul;65(7):2039–50.
5. Gelaye B, Clish CB, Denis M, Larrabure G, Tadesse MG, Deik A, et al. Metabolomics signatures associated with an oral glucose challenge in pregnant women. Diabetes Metab. 2019;45(1):39–46.
6. Ho JE, Larson MG, Vasan RS, Ghorbani A, Cheng S, Rhee EP, et al. Metabolite Profiles During Oral Glucose Challenge. Diabetes. 2013 Aug;62(8):2689–98.
7. Bentley-Lewis R, Xiong G, Lee H, Yang A, Huynh J, Kim C. Metabolomic analysis reveals amino-acid responses to an oral glucose tolerance test in women with prior history of gestational diabetes mellitus. J Clin Transl Endocrinol. 2014 Apr 5;1(2):38–43.
8. Shaham O, Wei R, Wang TJ, Ricciardi C, Lewis GD, Vasan RS, et al. Metabolic profiling of the human response to a glucose challenge reveals distinct axes of insulin sensitivity. Mol Syst Biol. 2008 Aug 5;4:214.
9. Miki T, Lee EY, Eguchi A, Sakurai K, Sawabe Y, Yoshida T, et al. Accelerated oligosaccharide absorption and altered serum metabolites during oral glucose tolerance test in young Japanese with impaired glucose tolerance. J Diabetes Investig. 2018 May;9(3):512–21.
10. Lehmann R, Friedrich T, Krebiehl G, Sonntag D, Häring H-U, Fritsche A, et al. Metabolic profiles during an oral glucose tolerance test in pregnant women with and without gestational diabetes. Exp Clin Endocrinol Diabetes. 2015 Jul;123(7):483–438.
11. Wang TJ, Ngo D, Psychogios N, Dejam A, Larson MG, Vasan RS, et al. 2-Aminoadipic acid is a biomarker for diabetes risk. J Clin Invest. 2013 Oct 1;123(10):4309–17.
